# Graphene Oxide/Silver Nanoparticles Platforms for the Detection and Discrimination of Native and Fibrillar Lysozyme: A Combined QCM and SERS Approach

**DOI:** 10.3390/nano12040600

**Published:** 2022-02-10

**Authors:** Vania Tramonti, Cristiana Lofrumento, Maria Raffaella Martina, Giacomo Lucchesi, Gabriella Caminati

**Affiliations:** Department of Chemistry and CSGI, University of Florence, Via della Lastruccia 3-13, 50019 Sesto Fiorentino, Italy; vania.tramonti@gmail.com (V.T.); cristiana.lofrumento@unifi.it (C.L.); martinamariella@gmail.com (M.R.M.); giacomo.lucchesi@unifi.it (G.L.)

**Keywords:** graphene oxide, Ag nanoparticles, metasurfaces, QCM, SERS, lysozyme fibrils sensor, amyloid protein

## Abstract

We propose a sensing platform based on graphene oxide/silver nanoparticles arrays (GO/AgNPs) for the detection and discrimination of the native and toxic fibrillar forms of an amyloid-prone protein, lysozyme, by means of a combination of Quartz Crystal Microbalance (QCM) and Surface Enhanced Raman Scattering (SERS) measurements. The GO/AgNPs layer system was obtained by Langmuir-Blodgett assembly of the silver nanoparticles followed by controlled adsorption of GO sheets on the AgNPs array. The adsorption of native and fibrillar lysozyme was followed by means of QCM, the measurements provided the kinetics and the mechanism of adsorption as a function of protein concentration as well as the mass and thickness of the adsorbed protein on both nanoplatforms. The morphology of the protein layer was characterized by Confocal Laser Scanning Microscopy experiments on Thioflavine T-stained samples. SERS experiments performed on arrays of bare AgNPs and of GO coated AgNP after native, or fibrillar, lysozyme adsorption allowed for the discrimination of the native form and toxic fibrillar structure of lysozyme. Results from combined QCM/SERS studies indicate a general construction paradigm for an efficient sensing platform with high selectivity and low detection limit for native and amyloid lysozyme.

## 1. Introduction

Neurodegenerative disorders such as Parkinson’s and Alzheimer’s disease are chronic, degenerative pathologies that irreversibly damage the functional capabilities of the affected individuals. The common theme for all these pathologies is the neuronal misfunction and death flanked by the progressive loss of cognitive and motor abilities. Clinical studies associate these different neuropathies to different precursor proteins, or peptides, malfunctioning sharing a common pattern: the formation of misfolded fibrillar aggregates of protein, or oligomeric species, with high content of β-sheet secondary structure [1,2]. Such fibrillar aggregates eventually evolve in mature bundles of amyloid fibrils detected once the disease has proceeded to irreversibly impair the patient’s functions. Magnetic Resonance Imaging or Positron Annihilation tomography of the brain may evidence dark zones corresponding to cerebral damage in a stage too advanced to allow for remission. In recent years, it has become clear that amyloid begins accumulating in the brain decades before memory loss and major symptoms appear whereas growing consensus indicates that the crucial key point is the beginning of pharmacological interventions earlier in the disease process. Yet, robust and reliable diagnostic methods to detect the occurrence of low concentrations of toxic fibrils are still lacking, although extensive scientific efforts have been recently devoted to the identification of suitable detection systems [3,4].

The aim of the present work is the design, construction, and characterization of a novel sensing platform for an amyloid prone protein, i.e., lysozyme, used as a representative model of fibrillar aggregates to establish an efficient sensing method for the detection of early stages of neurodegenerations combining two powerful experimental techniques: Quartz Crystal Microbalance (QCM) and Surface Enhanced Raman Spectroscopy (SERS). With this approach, not only could we quantify the amount of lysozyme present in the solution, but we could also ascertain whether the QCM signal was due to the native form or to the toxic amyloid fibrils of lysozyme by careful examination of the specific SERS fingerprint of the protein secondary structure.

Lysozyme is a natural antibacterial protein ubiquitously distributed in diverse organisms including bacteria, viruses, plants, and animals that in its compact native globular form has a secondary structure consisting of 30% of α-helix structures, 64% random coil, and only a small fraction (6%) of β-sheets [5]. In acidic conditions and high temperatures, lysozyme secondary structure rapidly converts to amyloid fibrillar forms with high β-sheet content becoming a representative model of amyloid-prone proteins [2,6,7,8]. The detection of native lysozyme is important on its own right, for example, real-time monitoring of lysozyme concentration in biological fluids would act as an early warning for many diseases including bacterial infections, rheumatoid arthritis [9], breast cancer [10] leukemia, and kidney disorders [11]. Even more crucial is the detection of trace amounts of amyloid aggregates of lysozyme in biological fluids not only in connection with the early diagnosis of neurodegenerative diseases [12] but also for lysozyme amyloidosis [13]. This latter pathology, caused by the precipitation of lysozyme aggregates within the body, is characterized by multi-organ dysfunction, but its diagnosis still remains challenging [14,15].

Conventional methods for native lysozyme detection in solution, including electrochemical sensors [16], enzyme-linked immunosorbent assays [17], or molecularly imprinted film assays [18,19] require expensive antibodies and awkward experimental procedures. More recently, Pereira-Barros et al. proposed an aptamer-based Surface Plasmon Resonance method to selectively detect lysozyme concentrations as low as 0.5 μmol/L [20,21]. Lower detection limits were obtained using more complex procedures involving Fluorescence Energy Transfer [22], luminescent G-quadruplex Iridium(III) complexes [23], field-effect transistor (FET)-based sensing [24], and fluorescent biosensor coupled with gold nanoparticles [25,26]. On the other hand, studies on the detection of the fibrillar form of lysozyme are only in their infancy.

In the present work, we propose a sensing platform that detects and discriminates between native and fibrillar lysozyme based on a hybrid metasurface formed by silver nanoparticles and graphene oxide layers that can be coupled to both QCM and SERS detection systems. Our approach builds on previous experiments on a simpler prototype of this platform that revealed a superior analytical performance in the detection of small probe molecules such as Rhodamine G and alanine [27,28] by means of Surface Enhanced Raman Spectroscopy (SERS). 

SERS, proven to be an ultrasensitive label-free detection technique for a variety of chemical species [29,30], exploits the enhancement of the Raman signal of any molecule adsorbed on a metallic surface or located in the proximity of the metallic surface. The process proceeds via electromagnetic or chemical interactions [31] and occurs either on planar metallic surfaces with roughness at the nanometer scale or on metallic nanoparticles [32,33,34]. Large SERS effects are strictly dependent on the gap distance of adjacent nanoscale domains with a strongly enhanced local electromagnetic field [31] commonly termed ‘‘hot spots” [35]. In addition to the strong enhancement largely due to the excitation of Surface Plasmon Resonance (SPR), closely spaced arrays of metallic nanoparticles offer an additional sensing capability based on the Localized Surface Plasmon Resonance (LSPR) sensitivity to subtle changes in the refractive index of the surrounding molecular environment [36].

As a major barricade in SERS detection, it is often difficult to precisely control the number and density of hot spots [37]. Here, we propose as a SERS-active platform an ordered layer obtained from a combination of silver nanocubes and nanospheres in order to improve the surface packing of the nanoparticles while preserving a high density of hot spots on the surface. The nanoparticle arrays were prepared to transfer a floating condensed monolayer of nanoparticles from the air/water interface onto solid support using the Langmuir-Blodgett technique, a well-known method for the fine-tuning of the density of the nanoparticles at the water-air interface that is readily transferred onto the solid support [27]. 

SERS measurements of lysozyme immobilized on the nanoparticle arrays is expected to reveal the secondary structure of the protein discriminating the native globular form from the neurotoxic fibrillar structure rich in amyloid β-sheet motifs thanks to the change of vibrational intensities of the bands due to the peptide backbone modes, i.e., amide I and amide III bands [38,39] while ensuring protein conformational stability during the measurement [40].

Recently, SERS was proposed for the ultrasensitive determination of lysozyme using a four-way helical junction molecule probe for signal amplification or surfactant-assisted galvanic reaction [41,42,43] to amplify the signal but all these methods rely on the signal of a popular SERS probe, Rhodamine 6G, either free or attached to the plasmonic nanoparticles. In the present work, amplification of the SERS signal was accomplished by veiling the AgNPs array with a Graphene Oxide (GO) layer. GO has benefited manyfold in sensing applications: firstly, GO coating attracts reproducibly and homogeneously a large fraction of the molecules in the proximity of the hot spots thanks to the combination of a hydrophobic structure with oxygenated surface functional groups. The GO-lysozyme interaction is so strong that recently GO-lysozyme bio-conjugates have been proposed as nanocarriers for the targeted therapy of B-cell-mediated autoimmune diseases [44]. Secondly, the GO veil can be readily prepared by direct physisorption on the nanoparticles’ assembly with precise control of surface coverage and thickness as opposed to complex vapor deposition techniques often used for graphene. Moreover, GO is stable in an aqueous environment and does not alter the SERS spectrum of the analyte as found for graphene or metals while supplying the SERS signal with additional and remarkable uniformity.

Nevertheless, the quantitative determination of the protein fibrillar form is not an easy task with SERS measurements and only a few reports are currently found (see Table S4). To overcome this drawback, we combined SERS analysis with QCM investigation that allows for determining, through a calibration procedure, the amount of lysozyme fibrillar form in solution. 

QCM, a well-known nanogram mass sensing device, has been widely used in biological research [45] due to its superior performance, high sensitivity, and facile operation. QCM can detect cumulative effects in a non-invasive way with high sensitivity providing not only the kinetics and the mechanism of adsorption but also the mass and thickness of the adsorbed protein as a function of protein concentration in solution. QCM structural characterization was flanked by Phase Contrast Microscopy and Confocal Laser Scanning Microscopy experiments on ThT-stained AgNPs and GO-AgNC/NS arrays covered with a saturated layer of the protein to confirm the presence of either the native or fibrillar form of lysozyme in the samples.

Surface Enhanced Raman Spectroscopy (SERS) experiments were then performed on AgNC/NS and on the hybrid GO-AgNC/NS array after native, or fibrillar, lysozyme adsorption to obtain a description of the secondary structure of the protein. Principal Component Analysis (PCA) was carried out on the whole set of the collected SERS spectra, the analysis showed a significant data grouping allowing for the discrimination of the two different structures of lysozyme: native globular conformation and toxic fibrillar forms. Comparison with experiments carried out on bare silver nanoparticles evidenced a significant enhancement of fibrillar lysozyme adsorption and a higher amplification of the SERS signal in the presence of the graphene oxide coating. 

Taken together the results can be hopefully extended to other proteins heavily involved in the early stage of neurodegenerative syndromes as α-synuclein, tau-protein, or A-β peptides that share with lysozyme the same amyloid β-sheet motif in their fibrillar conformation.

## 2. Materials and Methods

### 2.1. Materials

Hen egg-white lysozyme (14,400 Da), HWL-N, Thioflavine T, ThT, chloroform, and methanol were purchased from Sigma (Milano, Italy). Ethylene Glycol (EG, ≥99%) was obtained from Scharlab (Milano, Italy). Sodium sulfide nonahydrate, PVP (Mw 55000), silver nitrate, GO solution (4 mg/mL) were obtained by Sigma Aldrich (Milano, Italy). All aqueous solutions were prepared using ultrapure Milli-Q water obtained from a Milli-Q set-up (Millipore pH = 5.6 at 20 °C). Suprasil quartz slides were purchased from Hellma (Muellheim, Germany), 5-MHz AT-cut gold-coated QCM sensors were provided by Biolin (Espoo, Finland).

### 2.2. Synthesis of AgNC/NS

AgNC/NS nanoparticles were obtained following a procedure previously described through a polyol synthesis, in the presence of poly(vinylpyrrolidone) (PVP) as a stabilizing agent [46]. Briefly, EG (10 mL) was placed into a flask and heated under magnetic stirring in an oil bath at 150 °C for 1 h under a nitrogen flow. Then, 0.175 mL of a 0.72 mg mL^−1^ sodium sulfide solution and 3.75 mL of a 20 mg mL^−1^ PVP solution in EG were subsequently added to the flask. The flask was thermostated for an additional 10 min until the temperature of 150 °C was again established. A silver nitrate solution (1.25 mL) in EG with a concentration of 48 mg mL^−1^ was added dropwise to the reaction flask at a rate of approximately 1 mL min^−1^. The reaction was stopped after 40 min by placing the flask in an ice bath and by adding 30 mL of acetone. Nanoparticles were then centrifuged at 10,000× *g* for 30 min and then dispersed in ethanol or chloroform by using an ultrasonic bath. The washing procedure was repeated at least three times to ensure the complete removal of the reagents. The resulting nanoparticles contained mainly 45 nm size nanocubes, a smaller contribution of Ag nanospheres, and a negligible fraction of irregular aggregates [27,47]. A typical TEM image obtained for the samples is reported in the Supporting Information (Figure S3) confirming the reproducibility of the synthetic procedure.

### 2.3. Preparation of Native and Fibrillar Lysozyme Solution

Native lysozyme, HWL-N, solution was freshly prepared in TRIS/HCl 10 mM buffer before each experiment. Lysozyme concentration was estimated from the absorption peak at 280 nm using ε = 37,980 mol cm^−1^ [48]. Lysozyme aggregation was achieved using an established protocol [49] incubating 100 μM native lysozyme stock solutions in HCl (pH = 2) in a glass flask at 60 °C. Aliquots of the incubated solutions were withdrawn at a different time interval and characterized using Thioflavine T assay [50], 60 μL of the ThT solution in HCl (pH = 2) were added before each measurement to ensure equilibrium distribution of the probes. Final Thioflavine T concentration was 3 × 10^−6^ M in all samples. Fluorescence intensity of the monomer and dimer species of ThT spectra acquired along the progression of fibrillation is reported in Figure S1 of the Supporting Information and confirmed the presence of fibrillar lysozyme, HWL-F.

### 2.4. Photophysical Characterization

UV-Vis absorption spectra were recorded using a Lambda900 spectrophotometer (Perkin Elmer, Milano, Italy) with a 1 nm slit and 200 nm min^−1^ scan rate for solution samples. Absorption spectra of samples transferred on quartz slides were the average of 10 scans with 2 nm slit and 200 nm min^−1^ scan rate.

Fluorescence spectra were recorded on an LS50B spectrofluorimeter (Perkin Elmer, Milano, Italy), excitation and emission slits were set to 15 nm. QS cells with 1 cm optical path length from Hellma (Muellheim, Germany) were used for absorption and fluorescence measurements of solutions. All cuvettes and quartz slides were cleaned with piranha solution and carefully rinsed with water and ethanol. The cuvettes were dried by nitrogen flushing prior to each measurement. All fluorescence measurements were run at room temperature.

### 2.5. Circular Dichroism

Circular dichroism spectra were also run to ascertain the β-sheet content in fibrillar samples (see Figure S2 of the Supporting Information) before use. CD spectra were recorded using a J-715 Jasco spectropolarimeter with a 50 nm min^−1^ scan speed and data points were collected from 250 to 185 nm at room temperature with a 0.5 cm Hellma quartz cell or on Hellma quartz slides. Experiments were carried out in 10 mM TRIS/HCl buffer at pH 7.5. The protein concentration in the sample 1 mM. Random errors and noise were reduced for each spectrum acquiring at least 3 spectra. The signal acquired for the buffer was subtracted from the spectra recorded for the protein samples.

### 2.6. Preparation of Langmuir-Blodgett Films of AgNC/NS

A KSV3000 trough (KSV Instruments Ltd., Helsinki, Finland) filled with Milli-Q water (resistivity = 18 MΩ cm, pH = 5.6 at 20 °C) was used to prepare the Langmuir monolayers under symmetric compression. A total of 1.65 mL of a 3.1 mg mL^−1^ suspension of AgNC/NS in a chloroform/methanol (4:1 *v*/*v*) solution was dropwise deposited over the water surface and the monolayer was kept undisturbed for an additional 40 min to allow for complete solvent evaporation. Continuous spreading isotherms and hysteresis cycles were obtained using a barrier speed of 20 mm/min; surface pressure was measured with a platinum Wilhelmy plate as a function of the surface area at constant T = 20 °C. The temperature was controlled by means of a thermostatic bath (Haake, Berlin, Germany), accuracy in the temperature was 0.5 °C. The reported results are the average of at least three independent measurements. The shape and position of π-A isotherm strongly depend on factors such as the time allowed for solvent evaporation before starting compression, the amount of substance spread at the interface, and the compression speed. Careful optimization of these parameters generated reproducible isotherms up to π = 20 mN/m, compression beyond this value leads to unstable monolayers due to incipient collapse of the film and formation of 3D structures. Langmuir-Blodgett films were transferred onto quartz substrates by vertical dipping at a rate of 2 mm min^−1^, at the target surface pressure of π_tr_ = 15 mNm^−1^. Monolayers of AgNC/NS were transferred on Au-covered QCM quartz sensors with the same procedure. All substrates were cleaned with mild piranha solution, then carefully rinsed with ethanol, and treated in a plasma cleaner before LB transfer. To ensure reproducibility, four different substrates were dipped together into the trough well to be simultaneously coated by the same AgNC/NS layer.

### 2.7. Quartz Crystal Microbalance Measurements

QCM experiments were performed on a QCM-Z500 (KSV Instruments Ltd., Helsinki, Finland) with impedance monitoring equipped with a thermoelectric (TE) module (Oven Industries, Mechanicsburg, PA, USA) employing a gold-coated AT-cut 5 MHz quartz crystal (Biolin, Espoo, Finland) with frequency stability and resolution of ±0.05 Hz in water. The resonant frequency shift (Δf) and the change in energy dissipation (ΔD) were simultaneously measured at the fundamental resonant frequency (f_0_) and at five odd overtones (n = 3, 5, ..., 11), corresponding to resonance frequencies of f_n_ ≈ 5, 15, 25, 35, 55 MHz. The measuring cell was kept at T = 20.0 ± 0.1 °C with a Peltier element connected to the TE module; room temperature was 22.0 ± 0.1 °C.

For thin, uniform, and rigid films in solution, the measured frequency shift Δf is linearly proportional to the mass density, Δm/A, of the deposited film according to the Sauerbrey Equation (1)

(1) $$\Delta f=-\frac{2nf_{0}^{2}}{\sqrt{\rho_{q}\mu_{q}}}\cdot \frac{\Delta m}{A}$$

where n is the overtone number and ρ_q_ (2.648 g/cm^3^) and µ_q_ (1011 g/cms^2^) are the density and the shear modulus of the quartz crystal, respectively [51]. For thicker or softer films, the resonance frequency is affected not only by the mass attached to the surface but also by the rigidity of the adsorbed layer since frictional losses occurring in the adsorbed layer may lead to a damping of the oscillation that depends on the viscoelastic properties of the material. Thickness and viscoelastic properties of the adsorbed film were obtained by means of the commercial software QCMBrowse (KSV Instrument Ltd., Helsinky, Finland) fitting the data to a two-layer model that relates the measured Δf and ΔD of all overtones, to the viscoelastic properties of the adsorbed layer and the surrounding solution using a Voigt-based model previously used for similar systems [52,53].

### 2.8. Confocal Laser Scanning Microscopy (CLSM) and Phase Contrast Microscopy

Imaging of ThT-stained lysozyme adsorbed on the nanostructured platform was performed by confocal microscopy (Leica Microsystems, Heidelberg, Germany) in inverted configuration (DM IRE2) with head CT scan SP2, and electronic control unit CTR MIC Box. All images were scanned using a 63× objective for immersion in water and an Argon laser for the 458 nm excitation line.

Phase contrast images were obtained with a Nikon Diaphot 300 Fluorescence Phase Contrast Inverted Microscope (Tokyo, Japan).

### 2.9. SERS Measurements

Raman measurements were performed at room temperature on RENISHAW RM2000 micro-Raman apparatus (Pianezza, Italy) with a 785 nm laser as excitation source unless otherwise stated in the text. The relatively lower photon energy of the 785 nm laser was chosen to avoid thermal degradation of the protein. We used a 50× objective with accumulation times of 30 s per spectrum and a 70 µW power on the sample. The accumulation times and the laser power were the same for all Raman measurements.

SERS measurements were performed directly on AgNC/NS and GO-AgNC/NS coated QMC sensors after monitoring the adsorption of native Hen egg-white lysozyme (HWL-N) or fibrillar Hen egg-white lysozyme (HWL-F).

In the case of SERS measurements on glass substrates coated with GO-AgNC/NS layers, the coated slides were immersed in HWL-N or HWL-F solution for two hours to ensure that adsorption equilibrium was reached, then repeatedly rinsed with buffer and dried under nitrogen flux before each SERS measurement.

## 3. Results and Discussion

### 3.1. Fabrication of the GO-AgNP Sensor Platform

**Figure 1:**
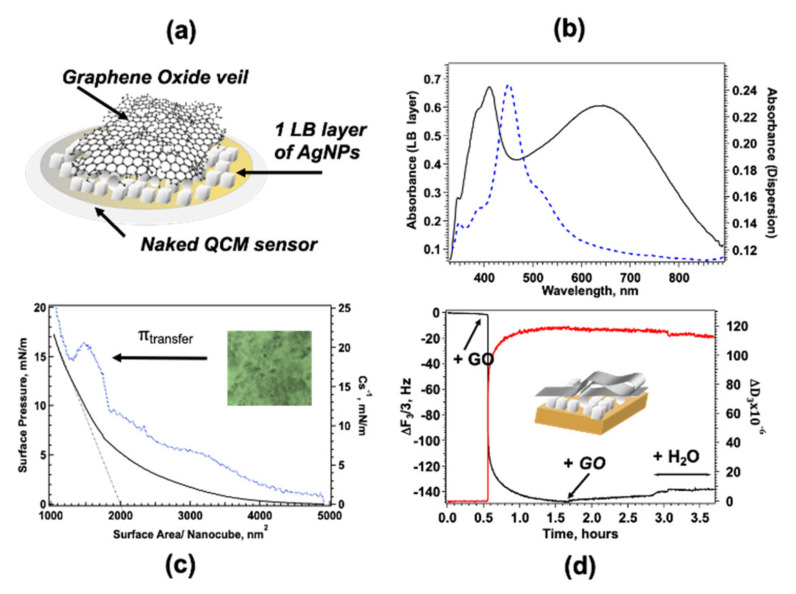
(**a**) Cartoon of the layered GO-AgNC/NS structure built on the QCM sensor. (**b**) Absorption spectra of AgNC/NS dispersion (blue dashed line) and of 1 LB layer of AgNC/NS transferred at 15 mN/m (black solid line). (**c**) Surface pressure-area C_s_^−1^-A isotherm for a monolayer of AgNC/NS at water-air interface; inset: optical microscopy image in reflection mode of a single AgNC/NS monolayer. (**d**) GO adsorption on AgNC/NS monitored by QCM.

SERS enhancement with uniform distribution of the analyte requires a series of carefully devised and realized steps: a cartoon of the nanostructured platform composed of AgNC/NS veiled with GO leaflets is reported in Figure 1a. The proposed architecture is obtained by sequential transfer of a compact array of AgNC/NS on solid support followed by adsorption of GO platelets. The procedure involves two distinct steps reported here separately: fabrication of an ordered array of Ag nanoparticles and coating of the nanoparticle monolayer with graphene oxide. GO-AgNC/NS architectures were obtained either on Ag coated quartz crystals for combined QCM and SERS measurements, the same procedure was used on quartz substrates for optical characterizations and control SERS experiments. The AgNC/NS dispersion was preliminarily characterized by UV-Vis spectroscopy (Figure 1b) that evidenced a sharp peak around 450 nm due to the Localized Surface Plasmon Resonance (LSPR) of the nanocubes and a small shoulder at 510 nm. According to previous reports, the position of the LSPR band marks the presence of nanocubes with edge size close to 50 nm in agreement with TEM (Figure S3) and AFM [28] results. Minor peaks (348 and 380 nm) are likely due to the small population of Ag nanospheres and nanoparticles with different morphology as reported earlier [54].

#### 3.1.1. Fabrication of Ag Nanoparticle Layers with Fine Tuning of Nanoparticles Surface Density

Compact arrays of silver nanoparticles were obtained by the Langmuir-Blodgett (LB) technique, this choice was supported by previous work from this group [28] that selected the LB transfer as the optimal procedure to produce reproducible, SERS-active substrates with a large fraction of AgNC/NS clusters with a small interparticle distance that permits the creation of efficient distribution of hot-spots. In addition, measurements of localized surface plasmon emission via delayed femtosecond laser pulses confirmed that small clusters lead to a plasmonic response that provides the highest peak intensity [55].

PVP capped silver nanoparticles were spread at the water-air interface from a chloroform dispersion in a Langmuir trough to fabricate a monolayer of Ag nanoparticles. In agreement with recent works from this group [28] we found that AgNC/NS easily spread at the water air-interface giving ordered and stable floating monolayers, a typical π-A isotherm for AgNC/NS is reported in Figure 1c as a function of surface area. Surface pressure increases monotonically as the available surface area decreases showing a subtle phase transition at π = 4 mN/m in agreement with previous findings [28]. The surface compressional modulus was computed from the experimental π-A data using Equation (2).

C_s_^−1^ = −A(dπ/dA) (2)

The surface compressional modulus, related to the elasticity and fluidity of the monolayer [28] identifies the different monolayer phases with different rigidity. The spreading isotherms of Figure 1a shows a series of different packing transition marked by discontinuity in the Cs^−1^ which were not present in the compression of monolayers of Ag nanocubes [21]. The low value of the maximum Cs^−1^ indicates that the nanoparticles form a fluid and elastic monolayer, similar low values of the surface compressional modulus (Cs^−1^ < 50 mN/m] were found for nanoparticles trapped at the air–water interface also by other authors [56].

Compression-expansion cycles were performed on AgNC/NS monolayers arresting compression below 20 mN/m, the results showed only a negligible hysteresis that vanished completely after the second cycle. These findings exclude loss of material in the subphase upon compression and support the formation of an elastic array of AgNC/NS that quickly recover their closely packed morphology after expansion thanks to the presence of the PVP polymer surrounding the nanoparticles. Such features are essential to warrant a successful transfer of the film from the water subphase to the solid support when a fast re-organization of the nanocubes at the interface upon transfer to the solid substrate is needed. Langmuir-Blodgett layers were transferred onto glass and Au coated quartz supports at π = 15 mN/m after two expansions-compression cycles. We always obtained stable values of surface pressure along the transfer process and transfer ratios close to one (TR = 0.98 ± 0.10) over the entire surface of the solid support indicating an optimal quality of the transferred layer [57], a result likely due to the stabilizing effect of the PVP cushion capping the surface of the nanoparticles. Formation of compact arrays of nanoparticles through monolayer compression by the Langmuir-Blodgett technique is facilitated by the presence of small quantities of nanospheres that easily slide and accommodate in the interstitial spaces among the AgNC clusters.

Optical microscopy in reflection mode of LB monolayers of AgNC/NS transferred at 15 mN/m on quartz slides are reported in the inset of Figure 1c, the image shows a near-continuous and dense monolayer of nanoparticles with the presence of some 3D clusters of nanoparticles probably due to incipient monolayer collapse at this surface pressure.

A typical extinction spectrum of 1 LB layer transferred on quartz at 15 mN/m is reported in Figure 1b and reveals a peak at 412 nm and a broader band centered at 640 nm. The strong peak is ascribed to the blue-shifted intense quadrupolar resonance expected for arrays of nanocubes [58], in previous studies on LB arrays of DOPC/AgNC/NS of similar sizes [59] the authors assigned the signal observed at 414 nm to quadrupolar coupling modes and observed a red-shifted dipolar contribution. In our case the dipolar peak assignment is hindered by the superposition of the signal of non-cubic aggregates. More importantly, we observed a broad intense band due to strong interparticle dipole-dipole coupling centered at 640 nm, experimental and theoretical studies [60] on two dimensional (2D) arrays of AgNPs with the different edge-to-edge distances showed that delocalized long range LSPR results in a broad band centered 640 nm for interparticle distance d = 3 nm and that the band red-shifts with increasing d.

Monolayers of AgNC/NS were transferred also on naked QCM sensors, from the change in frequency of the piezoelectric sensor measured before and after the LB transfer we estimated the mass of the transferred AgNC/NS applying the Sauerbrey equation (Equation (1)), the results showed an excellent reproducibility of the LB procedure and provided an average value of the nanocubes surface density that translates in an average distance between single particles over the entire sensor surface of 10 nm. Similar values were obtained also for an LB layer of AgNC/NS [28] of similar size although local interparticle distance estimated by AFM evidenced much smaller gaps of 1–3 nm between face-to-face 50 nm thick nanocubes clustered in separated aggregates.

#### 3.1.2. Veiling the AgNP Arrays with Graphene Oxide

The GO-AgNP platform was carefully constructed veiling with Graphene Oxides on the surface of one LB layer of silver nanoparticles, this procedure was already proven successful in providing a uniform coating of GO leaflets on the nanocubes [28]. The precise control of GO architecture and its influence on the distribution of the silver nanoparticles is of crucial importance for the realization of efficient and reproducible SERS sensors where GO coating should assure a homogenous signal while amplifying the SERS effect.

Briefly, the physisorption of GO on AgNC/NS arrays deposited on a silver-coated QCM sensor was monitored recording the change of the normalized frequency of the third overtone, Δf_3_/3, and dissipation, ΔD_3_, as a function of time as shown in Figure 1d. Measurements were performed at 20 °C using [GO] = 400 mg L^−1^, previous works [29] demonstrated that such concentration ensures saturation adsorption. According to Sauerbrey Equation (1), a decrease in Δf_3_/3 corresponds proportionally to an increase in the GO mass adsorbed on the QCM sensor, such increase is flanked by an increase in ΔD_3_ that reflects an increase in elasticity of the adsorbed layer. Interestingly, the change of Δf_3_/3 e ΔD_3_ in time follows two different kinetic regimes likely associated with a first adsorption step followed by a slower reorganization of GO at the surface.

This behavior reflects a first adsorption step driven mainly by the hydrophobic interactions between the hydrocarbon backbone of PVP with the sp^2^ domains of GO, after a first surface anchoring the self-assembly process of GO is regulated by the competition between the repulsive electrostatic forces exerted by the negative carboxylic groups on the GO surface and the van der Waals attraction between graphene oxide sheets due to π-π staking. At low surface density repulsive electrostatic force due to negatively charged carboxylic groups prevail but as GO flakes accumulate in the boundary layer van der Waals attraction guides a dense packing of GO at the surface [61,62]. Interestingly, the increase in dissipation factor, ΔD_3_, indicates that GO is not adsorbed flat as a rigid monolayer, but that partly folded GO sheets cover the surface as also shown by AFM measurements on similar samples [29]. The high flexibility of graphene oxide sheets favors the interactions between the hydrophobic domains and could be responsible for crumpling, a behavior reminiscent of crumples GO phases reported also by other authors [63].

Surface saturation with GO is reached after 60 min as shown by Δf_3_/3 and ΔD_3_ behavior (see Figure 1d), the results also show how water-rinsing induces only a small decrease in the adsorbed mass without significant detachment of GO flakes from the surface confirming that GO is firmly adsorbed on the AgNC/NS array. Saturation corresponds to the maximum quantity of GO that can adsorb on the AgNC/NS array, further coating is hindered by electrostatic repulsion between partly folded flakes that can be easily removed by water rinsing. Similarly, veiling of the AgNC/NSs transferred on quartz slides for SERS, Phase Contrast Microscopy and CLSM measurements were obtained by direct immersion in a 400 mg L^−1^ aqueous dispersion of GO followed by water rinsing. The UV-Vis spectrum (see Figure S4 in the Supporting Material) shows the characteristic band centered at 229 nm, assigned to the layer of physiosorbed GO flakes [64], while the signal due to the plasmonic band of the nanoparticle, not observable in this scale, remains unaltered.

### 3.2. Comparative Adsorption of Fibrillar and Native Lysozyme on AgNC/NS and GO- AgNC/NS Monitored by QCM

The nanosensor platform prepared on the QCM sensor described above was used to study the adsorption and capture of both native globular lysozyme, HWL-N, and fibrillar lysozyme, HWL-F. For each protein form, solutions at different protein concentrations were added directly in the QCM measuring chamber containing either the AgNC/NS or the GO-AgNC/NS coated sensor. The change in frequency and dissipation was recorded as a function of time for all overtones, once constant frequency values were reached, rinsing with buffer was performed to remove the protein not adsorbed at the surface.

**Figure 2:**
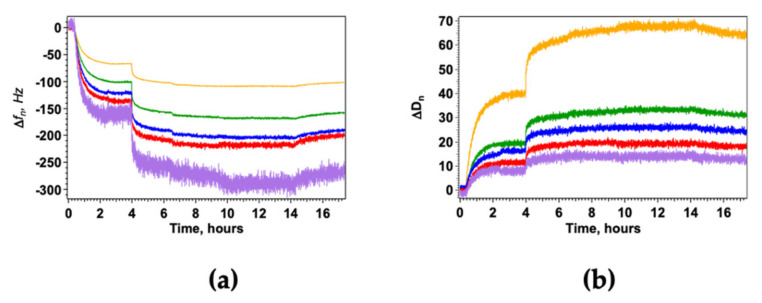
HWL-N adsorption on GO-AgNC/NS nanostructures monitored by QCM measurements. Δf_n_ (**a**) and ΔD_n_ (**b**) are the frequency shift and the dissipation factor for the fundamental frequency (yellow) and for the third (green), fifth (blue), seventh (red), and 11th (purple) overtones. The addition of increasing concentrations of HWL-N for 1 × 10^−8^ M < [HWL-N] < 1 × 10^−5^ M was followed by water-rinsing; temperature was kept constant at 20 °C for all measurements.

Typical results obtained for HWL-N are reported in Figure 2 but similar plots were obtained for all studied systems, the data clearly show a progressive decrease in Δf_n_ as HWL is added in the measuring chamber, flanked by an increased shift in dissipation that reveals the formation of an elastic adsorption layer of HWL-N and a high-affinity type adsorption [65]. Buffer rinsing evidenced only a minor decrease in the adsorbed mass allowing to exclude any release of silver ions observed by other authors [66] likely due to the protection of PVP molecules at the surface.

**Figure 3:**
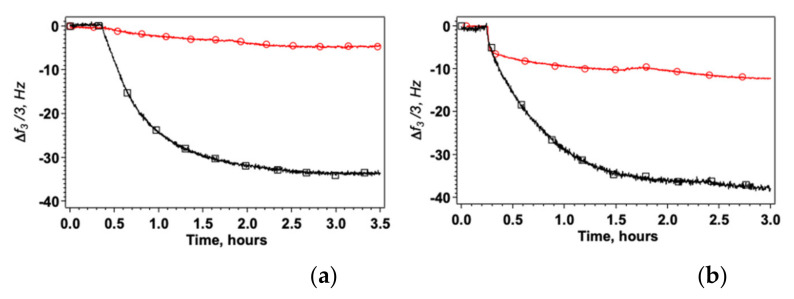
Change of Δf_3_/3 after addition of HWL-N (**a**) and HWL-F (**b**) to the nanostructured platform. Bare AgNC/NS arrays: red circles; GO veiled AgNC/NS arrays: black squares. Concentration of lysozyme = 1 × 10^−6^ M in all cases.

The measurements were performed using differently coated QCM sensors: AgNC/NS arrays and GO-AgNC/NS meta-structures exploring the same range of concentrations of lysozyme in solution. We observed that the presence of GO highly enhances the adsorption of both forms of lysozyme as shown in Figure 3, furthermore the kinetics of adsorption clearly indicate that the process is faster and more homogeneous in the presence of a GO veil.

Figure 3 shows that the amount of HWL adsorbed on GO veiled surfaces is always much larger compared to bare AgNC/NS arrays both in the case of the fibrillar and of the native form. Interestingly also, the kinetics of adsorption is markedly different for the two HWL forms as shown in Figure S5 in the Supporting Material. In the case of GO veiled samples, we always found a single exponential behavior corresponding to diffusion and adsorption on homogeneous surface sites. In contrast, when HWL-F adsorbs on AgNC/NS arrays, we observed two kinetic regimes: fast adsorption followed by a slow reorganization on the surface of the nanoparticle’s array (see Table S1). A similar time evolution of the spectra due to the conformational changes of the surface-adsorbed lysozyme with time was also found by Chandra et al. [67]. Control experiments using bare gold did not evidence the presence of significant quantities of HWL on the sensor confirming that the protein is interacting specifically with the capped nanostructures or with the GO sheets.

**Figure 4:**
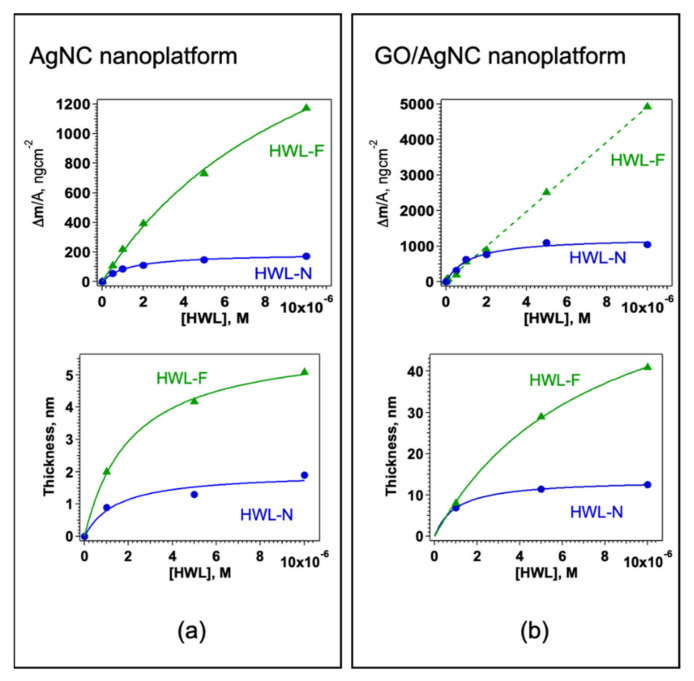
Surface density and thickness for HWL-N (green triangles) and HWL-F (blue circles) on (**a**) AgNC/NS and (**b**) GO-AgNC/NS platforms.

The adsorbed mass and thence the surface density for each HWL concentration was obtained from the normalized value of Δf_3_ at equilibrium, the resulting adsorption isotherms are reported in Figure 4 together with the QCM thickness, δ.

Both HWL-N and HWL-F were found to follow a Langmuir type adsorption with monolayer saturation on AgNC/NS arrays (Figure 4a) whereas, on GO-coated nanocubes, the behavior of the two HWL forms differs (Figure 4b): only HWL-N saturates the surface at monolayer coverage whereas HWL-F density is continuously increasing with increasing solution concentration.

The values of the surface density of the native and fibrillar forms of HWL adsorbed at equilibrium versus protein concentration shown in Figure 4 were fitted using a Langmuir adsorption model described in Equation (3)

(3) $$\frac{\Delta m}{A}={\left(\frac{\Delta m}{A}\right)}_{Sat}\times \frac{K_{ads}\left[HWL\right]}{1+K_{ads}\left[HWL\right]}$$

where Δm/A_sat_ is the HWL surface density at saturation, i.e., at monolayer coverage, and K_ads_ is the adsorption constant. The molecular area occupied by HWL-N at surface saturation was obtained on the surface, A_mon_, obtaining from Equation (4)

(4) Amon=MWHWLNAΔmASat

where MW_HWL_ is the protein molecular weight, (Δm/A)_Sat_ is the surface density at monolayer coverage obtained from the Langmuir fit and N_A_ is the Avogadro number. In the case of HWL-F, molecular areas could not be calculated since the effective molecular weight of an average fibril cannot be determined.

For all the examined systems, the structural and energetic parameters obtained from the fit are shown in Table 1 together with the experimental thickness determined from the analysis of all the harmonics obtained from QCM measurements.

The data evidence that HWL-N adsorption leads to a different conformation of the protein at the surface depending on the outer layer: for naked AgNC/NS system the molecule forms layers with a smaller thickness (1.9 nm) and larger K_ads_ and ΔG_ads_ whereas when GO veils the nanoparticles the thickness increases and the surface area decrease resulting in a larger mass adsorption.

Our findings on the adsorption behavior on AgNC/NS are supported by previous adsorption studies of HWL-N onto negatively charged silica using dual-polarization interferometry (DPI) [68]. Lysozyme was observed to adsorb from sparse monolayer to multilayer coverage although the authors observed that the multilayer structure was easily removed by rinsing with a protein-free buffer solution leaving only a single monolayer of protein.

Previous crystallographic studies have shown that native lysozyme has an ellipsoidal shape with dimension 45 × 30 × 30 Å [69] (see Figure S6) and previous adsorption studies suggest that HWL-N may adsorb in at least two possible orientations, namely the ‘side-on’ where the long axis of lysozyme is parallel to the surface and the ‘end-on’ orientation where the long axis of lysozyme is perpendicular to the surface. Lysozyme can as well adsorb in between side-on and end-on orientations where the long axis of lysozyme and the surface form an angle, referred to as the ‘edge-on’ orientation [70]. Moreover, due to the higher height-to-width ratio and therefore larger interstices between the protein and the surface, the edge-on and the end-on orientations would be expected to show higher water solvation [71] and therefore larger QCM thickness.

After buffer-rinsing we observed HWL-N conformations corresponding exclusively to the slightly deformed side-on orientation of the protein with a thickness at monolayer coverage of ca. 2 nm as also reported by other authors [72] for AgNP-lysozyme bio-conjugated for SERS application. In contrast to previous studies on HWL adsorption on flat metal surfaces [73], we did not observe an edge-on adsorption even at low surface coverage, a difference evidently due to the presence of discrete nanoparticles or nanoparticle clusters and to the presence of the PVP capping agent. In the case of bare silver surfaces, the main driving force to HWL adsorption was identified in electrostatic interaction between the positively charged protein at neutral pH and the negative charge distribution of the silver surface, a feature partly screened by the presence of PVP. The entropy gain due to conformational changes is recognized to contribute to a very small extent to the driving force for HWL-N adsorption onto the silver surface since HWL-N—in contrast to “soft” proteins like albumin, immunoglobulin, and transferrin that show low internal conformation stability—is a small and rigid globular protein with a minor tendency for conformational changes upon surface adsorption [74].

The data in Table 1 also show that adsorption of HWL-N on GO covered surfaces is significantly larger than on Ag nanoparticles resulting in larger thickness and adsorbed mass, a behavior ascribed to the interplay of hydrophobic and electrostatic interaction between the protein and the hydrophobic and polar groups of the graphene oxide that may lead to multilayer coverage. Strong interaction between lysozyme and graphene oxide has been repeatedly reported in previous literature [75] and the general consensus is that the interaction forces results from the synergy of various forces including covalent interactions, based on the chemical reaction between the amino acid side groups and the functional groups available on the surface of graphene oxide [76], non-covalent adsorption through weak van der Waals forces, hydrophobic forces, π − π stacking interactions and electrostatic forces. The geometry of GO allows the hydrophobic region of GO to interact with the HWL hydrophobic region whereas the oxygen-containing functional groups of GO are responsible for hydrogen bonding to function [77].

A different picture emerges for the adsorption of the fibrillar form, the thickness of the adsorbed layer span from 5 nm on AgNC/NS to 41 nm onto GO veiled nanoparticles. Since the thickness of lysozyme of a single fibril is in the 2–3 nm [2] the data show that the fibrils are either folded on the surface in the former case or present as soft multilayered structures or bundles. Moreover, in the case of HWL-F due to the stable although the flexible structure of the fibrillar form, the large ΔG of adsorption is only marginally due to entropic reasons since the fibrillar forms are not expected to undergo conformational changes upon adsorption leaving the leading role in driving surface adsorption to electrostatic and hydrophobic interactions.

Although the interaction between GO and HWL has been widely studied, most of the literature uses Trp fluorescence quenching due to GO, or other spectroscopic methods, to determine GO-HWL dissociation constant in solution, in the present study we assessed a direct quantification of adsorbed lysozyme on the surface providing at the same time structural information on the adsorption layer.

### 3.3. Morphological Inspection with Phase Contrast Microscopy and Confocal Laser Scanning Microscopy Using ThT

The difference in the adsorption behavior observed for the native and the fibrillar forms of HWL was further investigated by direct imaging of the proteins adsorbed on AgNC/NS and GO-AgNC/NS prepared on quartz slides using Phase Contrast and Confocal Laser Scanning Microscopy using Thioflavine T, ThT, for fluorescent staining. ThT is well known to intercalate among the β-sheets of amyloid fibers giving rise to a specific emission at 480 nm upon excitation at 450 nm [8] due to the restricted rotational mobility sensed by ThT in the confined β-sheet environment and formation of closely spaced dimers. Scanning the surface of the sample containing HWL-F clearly evidenced the presence of fluorescent fibrillar aggregates. The images reported in Table 2 show interesting features on the morphology of the adsorbed layer and the peculiar localization of the fibrillar aggregates on the AgNC/NS arrays: the fibrillar bundles adsorb preferentially following the border of the AgNC/NS clusters, in contrast to the case of GO-AgNC/NS layer where the HWL-F fibrils are homogeneously and randomly adsorbed on the GO veil.

In the case of native HWL, CLSM did not evidence the fibrillar fingerprint on either of the two platforms but interestingly, if the native lysozyme sample is left aging on the AgNC/NS platform, a progressive transition to aggregated HWL-F forms is clearly observed (Figure S7) suggesting that the longer interaction of lysozyme with the Ag surface may lead to denaturation and aberrant misfolding of the native HWL.

### 3.4. Calibration Curve for Fibrillar HWL on GO-AgNC/NS

**Figure 5:**
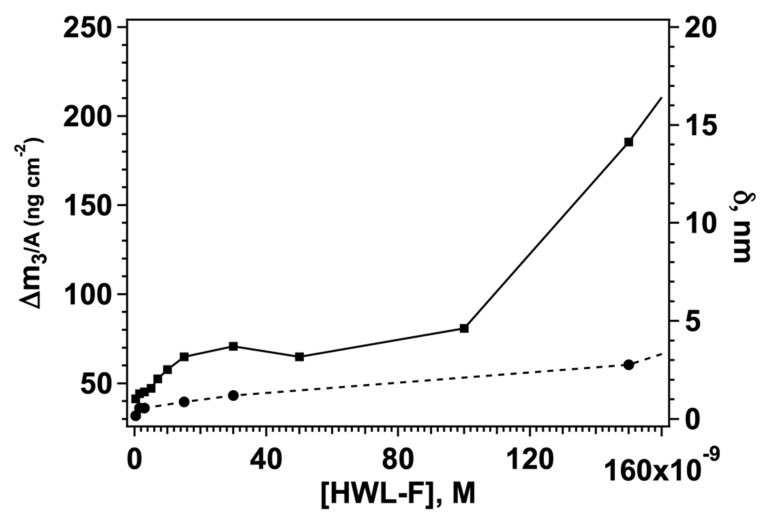
Adsorbed mass (solid line, left *y*-axis) and adsorbed layer thickness (dashed line, right *y*-axis) for HWL-F adsorbed on the GO-AgNC/NS platform.

Taken together the previous findings indicate the use of GO-AgNC/NS platforms may be efficiently used to sequester HWL-N and HWL-F forms from solution in a concentration-dependent manner. The adsorbed quantity can readily be quantified by QCM measurements paving the way for the use of these devices as sensors for both the native and the fibrillar forms of HWL. This result is particularly important for the detection of the fibrillar forms of the protein and may serve as a link to bridge the SERS signal coming from the nanoparticle array to the protein concentration in the original solution. We constructed a calibration curve for HWL-F measuring the QCM signal on GO-AgNC/NS covered sensors for a series of samples spiked with increasing quantities of HWL-F. The resulting adsorbed mass at equilibrium after buffer rinsing is reported in Figure 5 as a function of HWL-F concentration in solution for concentrations lower than 1.5 × 10^−8^ M.

In fact, for HWL-F concentrations lower than 100 nM, we observed a first adsorption saturation threshold with a layer thickness corresponding to a single fibril suggesting that a monolayer of extended fibrils is formed at these concentrations. The data in Figure 5 show the existence of a linearity interval between the mass of the protein on the GO-AgNC/NS array and the initial concentration of HWL-F in solution, a typical example of a calibration curve for this nanostructure is reported in Figure S8b together with the results of the linear regression fit (Table S2). In the low concentration regime, we found a linear correlation between the signal and [HWL-F], from the analysis of the calibration straight line we estimated the experimental limit of detection, LOD [78] for fibrillar lysozyme to be LOD = 1.97 nM. Recent literature on the analytical concentration detection of the fibrillar form of lysozyme reports LOD similar or higher than the value reported in this work (see Table S4).

For higher concentrations, the thickness increases above the value accepted for single fibril dimensions indicating that the adsorption layer is formed by multilayers of fibrils, or of entangled fibrils, as shown also in the CLSM and phase contrast microscope images (Table 2). Even though there is a correlation between adsorbed fibrillar mass and solution concentration also in this range (Figure S8), the results are less reproducible and dependent on the real “fibrillar concentration” in a solution that cannot be determined and varies according to the fibrillation process.

The mass-density to concentration curve for HWL-N also shows a linear portion before reaching monolayer saturation (Figure S8), in this case, the LOD reported in Table S2 is significantly larger (LOD = 14.8 nM). Although more complex sensing procedures have been shown to provide lower detection limits, many papers report LOD falling in our range of values (see Table S3) for similar clinical applications [79]. Considering that lysozyme levels can be significantly elevated to more than 1 or even 7 mM in patients suffering from leukemia, renal disease, and sarcoidosis the present results might be successfully used for HWL-N detection in body fluids.

### 3.5. Comparative SERS Study of Native and Fibrillar Lysozyme (1 × 10^−5^ M) on AgNC/NS and GO-AgNC/NS Platforms

SERS experiments were performed on the same QCM samples of native and fibrillar lysozyme adsorbed on AgNC/NS and GO-AgNC/NS arrays to obtain a description of protein conformation focusing on the spectral features and β-sheet content that allows for discriminating between the HWL-F from HWL-N. Changes in protein structures induced by the fibrillation process can be readily monitored using vibrational spectroscopy monitoring the occurrence of the amide I band corresponding to organized β-sheet structure present in amyloid fibrils [80], Raman scattering is highly sensitive to changes to the conformational changes of amino acid residues, as well as to the variations of the aromatic functional groups and most importantly of the amide groups that directly correlate to the secondary structure of proteins [81,82]. The amide I band with a major contribution of C=O stretching vibration is generally observed ranging between 1630 and 1700 cm^−1^, whereas amide II (N–H bending coupled with C–N stretching) and amide III (C–N stretching mixed with N–H bending vibration) are observed in the range between 1530–1580 and 1220–1300 cm^−1^, respectively.

Lysozyme is a globular protein that contains 129 amino acid residues of which three are Phenylalanine (Phe), three Tyrosine (Tyr), six Tryptophan (Trp), and one Histidine (His), the bands due to the aromatic side groups are expected to be strong and their relative intensities when HWL adsorbs on the silver surface reflect the state of adsorption on the silver colloid as the vibrational modes lying close to the surface are preferentially enhanced than those extending far apart [80].

The amide bands in HWL consist of mainly three structural components—three stretches of α-helix, antiparallel pleated β sheet, and random coils, which are folded in an irregular way as shown in Figure S6. The positions and intensities of the SERS signal for the vibrational peptide backbone modes, i.e., amide I and III bands, can be used for an empirical estimate of the protein secondary structures, in particular the contributions of α-helix, β-sheet, and random coil for the amide I are expected to occur over the spectral windows 1640–1658 cm^−1^, 1665-1680 cm^−1^, and 1660–1665 cm^−1^, respectively [83,84]. The amide III band extends over the spectral range between 1230 and 1310 cm^−1^ with the spectral peaks of α-helix, β-sheet, and random coils units located in the range 1260–1320 cm^−1^, 1235–1242 cm^−1^, and 1250–1260 cm^−1^, respectively [85]; the region 1254–1260 cm−^1^ has been assigned to β-turn. A summary of the major peaks for normal Raman of lysozyme in solution for both the native and fibrillar forms is reported in Table S5. Additionally, changes in position and intensity of SERS peaks provide information on the orientation of the molecule at the surface as well as on the group involved in chemisorption to the metal surface since SERS signals are preferentially detected from components closer to the surface [86].

**Figure 6:**
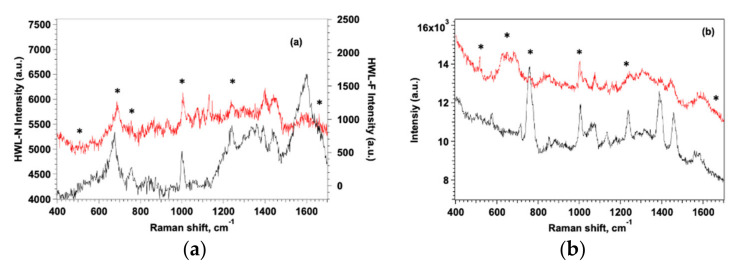
SERS spectra of HWL-N (black line) and HWL-F (red line) on AgNC/NS (**a**) and GO-AgNC/NC (**b**) platforms. [lysozyme] = 1 × 10^−6^ M. Distinctive peaks are marked by asterisks.

SERS measurements were performed using both quartz and gold as substrates for the metasurfaces, but we observed that the different nature of the support did not significantly affect the quality of SERS spectra although a better visual contrast was obtained for GO-AgNC/NS on quartz (Figure S9). SERS spectra recorded between 400 and 1700 cm^−1^ for HWL-N and HWL-F adsorbed on SERS AgNC/NS and GO-AgNC/NS substrates are reported in Figure 6.

It is evident in all graphs that the native lysozyme always presents higher intensity spectra on both platforms, since QCM data demonstrated a larger adsorption mass for the native form, this result confirms that HWL-N resides completely on the surface as a monolayer while HWL-F extends further away from AgNP hot-spots protruding into the solution. As expected, we observed that the presence of GO improves the sensitivity and the stability of the signal protecting AgNC/NS from oxidation but more importantly veiling the nanoparticles array with GO notably enhances the intensity and the uniformity of the SERS signal.

The spectra in Figure 6 also reveal substantial differences between HWL-N and HWL-F and evidence the role of GO coating in amplifying such differences although the presence of signal due to GO may in some regions render the interpretation ambiguous. GO is known to present two main SERS bands located at 1351 cm^−1^ and 1590 cm^−1^ corresponding to the D and G bands, respectively. The D band represents disordered sp3 carbon while the G band is associated with the stretching mode of ordered sp2 crystalline graphite-like structures [22]. The spectra of Figure 6b were tentatively normalized to the G band of GO at 1580 cm^−1^ but the analysis of the normalized spectra provided substantially the same results.

The contribution to the intensity of the peak at 518 cm^−1^ comes from the four pairs of S-S bonds present in lysozyme: two of them are situated near the α-helix regions, while the other two are near the β-sheet regions. This suggests that the low intensity of this peak in both lysozyme forms could be due to a fraction of the S-S bonds which are not adsorbed, nor close to the substrate surface. In the case of HWL-F, the intensity is even lower suggesting that the Cys groups are located even further away from the surface with distortion of the dihedral angles with respect to the native form. Podstawka et al. [87] who utilized Raman and SERS to investigate the adsorption of proteins containing disulfide bonds onto colloidal silver surfaces, revealed that bonding to the silver surface takes place at disulfide bridges located between Cys(5)-Cys(127) and Cys(30)-Cys(115) in HWL-N without any cleavage of the S–S bonds. The same study confirmed our results showing that HWL-N did not undergo any denaturation or large conformational change upon bonding to the colloidal silver surface since both the amide I and amide II peaks persist in the SERS spectra.

The sharp band at about 1005 cm^−1^ of the symmetric ring-breathing vibration mode is present in all SERS spectra but the peak is always more intense in the case of HWL-F suggesting that the benzene ring adopts a standing up geometry or at least a tilting one if considering the surface plane in this case. When this band is missing or weaker, as found for HWL-N, the benzene ring lies almost flat on the surface [41]. Trp orientation might be similar to Phe, as is shown by the presence of a strong 1015 cm ^−1^ band.

SERS spectra in Figure 6 evidence the distinctive peaks of HWL-N and HWL-F (marked with asterisks), all spectra show bands that originate mainly from the aromatic amino acids, i.e., Tyr, Trp, His, and Phe in the 630–720 cm^−1^ range, the intensities of the bands associated with aromatic amino acids Phe and Tyr and with the stretching vibration C-S are of similar intensities on AgNC/NS.

SERS spectra clearly show the importance of the GO addition in revealing the presence of small concentrations of lysozyme, thanks to the Tryptophan-Indole breathing band (763 cm^−1^) and the Phenylalanine ring breathing band (1004 cm^−1^).

The presence of strong vibrational modes of Trp and Phe residues in the SERS spectra of lysozyme indicates that both residues are close to the Ag surface occupying adjacent sites in the adsorbed molecule. Further support for the localization of Tyr residues in HWL-N close to the surface is provided by the presence of a doublet in the 800–850 cm^−1^.

Therefore, it is most likely that the lysozyme molecules are adsorbed on the Ag surface via the region marked in Figure S6, closer to the α-helix region which includes both Trp-123 and Phe-34 residues of the molecule as proposed also by other authors [41,88]. In the case of GO veiled nanoparticles, the intensities of these bands are much larger for HWL-N than for HWL-F indicating a preferential interaction of these amino acids with the surface in the case of the native form.

More importantly, we observed that the peak at 1239 cm^−1^ due to the amide III contribution of organized β-sheet is detected with much higher intensity for the fibrillar HWL-F form compared to the HWL-N case; also, in this case the presence of GO provides larger amplification of the signal.

Interestingly, the peak at 758 cm^−1^ due to the indole ring breathing mode of the Trp residue is always more intense for the fibrillar form and the effect is even amplified on GO-covered sensors suggesting that this residue is located closer to the surface when the protein is in a fibrillar form and in an environment of lower hydrophobicity compared to naked AgNP where the intensity of the signal was much smaller [40].

Taken together the results show that the aromatic amino acid residues sidechains Phe, Cys, and Tyr are close to the sensor surface in the case of HWL-N, while Trp is the main active protein site directly in contact with the substrate surface in the case of HWL-F.

The native and the fibrillar forms can also be easily discriminated in the 1200–1600 cm^−1^ spectral range by the presence of the peak at 1398 cm^−1^ assigned to the adsorption of carboxylate groups [89] of the amide I: the intensity of this group is larger for HWL-F than for HWL-N especially when the protein is adsorbed on GO-AgNC/NS.

Although in the spectra with excitation at 785 nm reported in Figure 6, the amide I band typical of β-sheet structures of the fibrillar form is only present as a shoulder at 1666 cm^−1^, SESR spectra obtained with 532 nm excitation evidenced the peak much more clearly for the HWL-F adsorbed on GO-AgNC/NS as shown in Figure S10.

Interesting information was obtained also from SERS spectra in the range 180–400 cm^−1^ reported for all systems in Figure S11. Vibrations in this spectral range refer to the bonds involving the Ag atoms, previous literature reports SERS bands at 220 cm^−1^, 238 cm^−1^, and 243 cm^−1^ for Ag-O, Ag-N, and Ag-S, respectively [67,89,90]. SERS spectra evidence a Raman shift for these typical bands as shown in Table 3; the assignment of an intense and sharp peak at 235 cm^−1^ to Ag–N vibrations suggests the direct chemisorption of the proteins on the Ag-NPs surface as reported also by other authors [67,91]. In the case of HWL-N on GO-coated AgNP arrays, the same signal is present although superimposed to the Ag-O signal at 227 cm^−1^ due to the interaction of silver with the oxygen-containing groups of GO. In principle, a contribution to the Ag-O bands may derive also from residual PVP that binds to the AgNC surface through the oxygen atom in the amide group of PVP [92]. Nevertheless, such contribution is probably negligible since previous studies have shown that PVP is massively displaced from the surface both by protein adsorption and by copious rinsing [93]. Furthermore, there is no band stretching in the 1500 cm^−1^ and 1700 cm^−1^ range which is the typical feature of the C-N and C=O vibrations of PVP molecules [94].

For HWL-F on naked AgNP, the spectrum shows a shoulder at 234 cm^−1^ and a peak corresponding to Ag-S interactions suggesting a change in the interaction of Ag with the functional groups of the protein and a change in the orientation of the adsorbed species. The effect is even stronger for GO coated samples and the data describe changes in the surface interaction site with HWL-F, the fibrillar form is adsorbed with the Cys groups exposed and closer to the interface. Recent fluorescence quenching studies also support this hypothesis suggesting that lysozyme and GO form a stable complex followed by conformational changes of the protein [95].

Although the most evident spectral differences are due to the aromatic side groups adsorbed on the sensor surface, experiments as a function of the excitation laser line allowed to reveal even more clearly the fingerprint of an organized organization of β-sheet corresponding to fibrillar lysozyme in the 1660 cm^−1^ amide I band (see Figure S10) [96].

**Figure 7:**
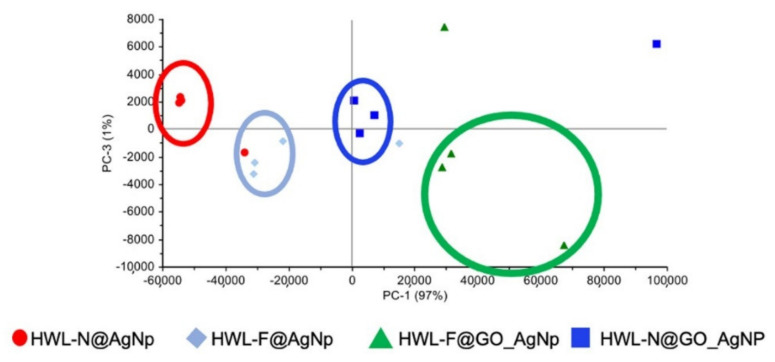
Scores plot of the whole collected SERS spectra.

Preliminary Principal Component Analysis (PCA) was performed on a set of SERS spectra collected for different excitation wavelengths for GO-AgNC/NS nanosensors considering the spectral range 200–2000 cm^−1^. The analysis of these preliminary results showed a significant data grouping (Figure 7), which was ascribed to different structures of lysozyme and/or the presence/absence of GO on the surface of the sensor substrate.

Briefly, the scores plot referred to this first set of samples and shows a satisfactory grouping of the spectra essentially on the PC-1 axis, for which the major contribution arises from the spectral shift of the band in the 200–250 cm^−1^ range, typical of the interaction between Ag and O, N or S. The information from the loading plot of PC1 evidence the trend towards higher wavenumbers along the series GO-HWL-F, GO-HWL-N, HWL-F, and HWL-N, suggesting both a stronger interaction between the protein and the AgNPs due to the presence of GO and in general a more stable interaction of HWL-F with respect to HWL-N.

The outline of PC-3 in the loading plot shows spectral variations in the range attributable to amide I group (1660–1670 cm^−1^) evidence discrimination due to the formation of β-sheet structures.

We also exclusively analyzed the 380–2000 cm^−1^ range eliminating from the PCA calculation the major contribution to the variability of the data comprised in the range 200–380 cm^−1^ to better evaluate subtle differences. The results distinctly demonstrated a higher enhancement in presence of GO especially HWL-F.

The differentiation of the probe molecules based on the peaks in the 200–250 cm^−1^ region has not been studied in the literature, even if it could represent an advantageous tool for fine discrimination of probe molecules and it will be faced in a specific study. In the present work, this step of analysis is proof of the superior SERS efficiency when using GO and it is a proof-of-principle that shows the possibility to spectrally discriminate HWL-F from HWL-N also in the low wavenumber region.

## 4. Conclusions

The body of the results shows that the combination of QCM and SERS measurements allows for the quantification of a model amyloid-prone protein, lysozyme, providing at the same time a way to discriminate between native conformation and fibrillar amyloid structures. We constructed a powerful capturing metasurface on the QCM sensor by Langmuir-Blodgett assembly of a mixture of AgNC/NS followed by the controlled adsorption of GO sheets on the AgNC/NS array. The procedure was found to be highly reproducible and adaptable to various solid substrates. The adsorption of native as well as fibrillar HWL from solution to the metasurface was followed by QCM and the data unveiled that the adsorption process proceeds with a Langmuir-type mechanism for both AgNC/NS and GO-AgNC/NS systems but with different maximum surface density at monolayer saturation, different adsorption constant K_ads_ and adlayer structure. More importantly, in the case of monolayers of single fibrils of HWL-F, a calibration curve was constructed with spiked samples correlating the concentration of HWL-F in bulk solution with the adsorbed quantity on the GO-AgNC/NS nanosensor. SERS spectra acquired on the QCM samples reflect the orientation of the protein layer and the difference in the secondary structure for HWL-N and HWL-F containing samples deriving from the presence of β-sheets in the fibrillar form.

Additional SERS signal amplification and improved signal uniformity were supplied by the GO coating, this allowed for the detection of small concentrations of lysozyme monitoring the Tryptophan–Indole breathing band and the Phenylalanine–ring breathing band. Together with the differences in the spectral region associated with the presence of β-sheet in the fibrillar form, a preliminary multivariate analysis approach in the low wavenumbers range was proved to be promising for the discrimination of the amyloid form of HWL. Although the determination of HWL-N is important in its own right, sensing the fibrillar forms of the proteins is crucial in connection with the diagnosis of many diseases that involve the presence of fibrillar aggregates in biological fluids. These results may hopefully be extended to other proteins such as α-synuclein, tau-protein, or A- β peptides that, sharing with HWL the same amyloid β-sheet motif in their fibrillar conformation, are considered as biomarkers of the onset of neurodegenerations.

## Figures and Tables

**Table 1:** Structural characterization of HWL-N and HWL-F adsorption on the nanosensor platforms obtained from the fit of the experimental data to the Langmuir model.

		QMC Data		Langmuir Model		
Protein	Nanoplatform	Saturation Surface Coverage, ng cm^−2^	Maximum Thickness, nm	A_mon_, Å^2^	K_ads_, M^−1^	ΔG^0^, kJ/mol
HWL-N	AgNC/NS	190	1.9	1258	7.6 × 10^6^	38.5
	GO-AgNC/NS	1249	12.5	192	8.6 × 10^5^	33.3
HWL-F	AgNC/NS	1174	5.1	--	8.9 × 10^4^	27.8
	GO-AgNC/NS	4918	40.9	--	--	--

**Table 2:** Phase contrast and Confocal Laser Scanning Microscopy images of HWL-F adsorbed on AgNC/NS and GO-AgNC/NS platforms.

	AgNC/NS		GO-AgNC/NS	
**HWL-F**	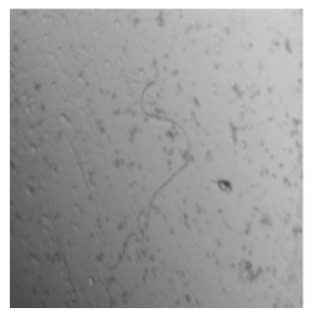	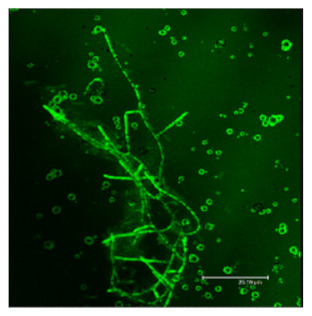	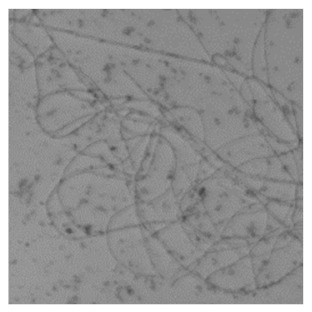	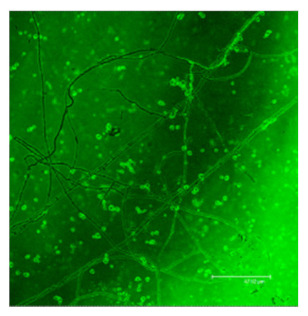

**Table 3:** Average position of SERS peaks in the 180–400 cm^−1^ range.

Protein	Peak Position, cm^−1^	Assignment	Peak Position, cm^−1^	Assignment
	AgNC/NS		GO-AgNC/NS	
HWL-N	235	Ag-N	226,238 sh	Ag-O, Ag-N
HWL-F	234 sh, 243	Ag-N, Ag-S	243	Ag-S

## Data Availability

All data supporting the findings of this study are provided within the paper and its Supplementary Materials. All additional information will be made available upon reasonable request to the authors.

## Supplementary Materials

The following supporting information can be downloaded at: https://www.mdpi.com/article/10.3390/nano12040600/s1, Figure S1: THT fluorescence emission as a function of time of incubation. Figure S2: Circular Dichroism spectra for lysozyme solution along the aggregation process. Figure S3: TEM images of the AgNC/NS nanoparticles. Figure S4: Absorption spectrum of GO-AgNC/NS metasurface prepared on quartz slides. Figure S5 (a) Kinetics of adsorption of HWL-N and HWL-F. Table S1. Fitting of the kinetic curves of adsorption. Figure S6: Native lysozyme conformation. Figure S7: Phase contrast (a) and Confocal Laser Scanning Microscopy (b) images. Figure S8: (a) Adsorbed surface mass density as a function of bulk concentration for spiked samples of HWL-N (a) and HWL-F (b) on GO-AgNC/NS sensors. Solid lines are the linear regression fit. (b) Calibration curve obtained for HWL-F for monolayers of fibrils (concentration regime 0–1.5 × 10^−8^ M). Table S2: Linear regression fit parameters and LOD. Table S3: Summary of relevant studies on native lysozyme detection. Table S4: Summary of relevant studies on fibrillar lysozyme detection. Figure S9: Optical microscopy in reflection mode of SERS samples. Table S5: Assignment of the main peaks observed in SERS spectra. Figure S10: SERS Spectrum for HWL-F adsorbed on GO-AgNC/NS, excitation at 532 nm. Figure S11: SERS spectra in the 180–300 cm^−1^ spectral range. References [97,98,99,100,101,102,103,104,105,106,107,108] are cited in the Supplementary Materials.

## References

[1] Knowles T.P.J., Vendruscolo M., Dobson C.M. (2014). The amyloid state and its association with protein misfolding diseases. Nat. Rev. Mol. Cell Biol..

[2] Chiti F., Dobson C.M. (2006). Protein misfolding, functional amyloid, and human disease. Annu. Rev. Biochem..

[3] Sheinerman K.S., Umansky S.R. (2013). Early detection of neurodegenerative diseases. Cell Cycle.

[4] Narayanaswami V., Dahl K., Bernard-Gauthier V., Josephson L., Cumming P., Vasdev N. (2018). Emerging PET Radiotracers and Targets for Imaging of Neuroinflammation in Neurodegenerative Diseases: Outlook beyond TSPO. Mol Imaging.

[5] Artymiuk P.J., Blake C.C. (1981). Refinement of human lysozyme at 1.5 A resolution analysis of non-bonded and hydrogen-bond interaction. J. Mol. Biol..

[6] Ow S.-Y., Dunstan D.E. (2013). The effect of concentration, temperature and stirring on hen egg white lysozyme amyloid formation. Soft Matter..

[7] Hill S.E., Miti T., Richmond T., Muschol M. (2011). Spatial Extent of Charge Repulsion Regulates Assembly Pathways for Lysozyme Amyloid Fibrils. PLoS ONE.

[8] Chaari A., Fahy C., Chevillot-Biraud A., Rholam M. (2015). Insights into Kinetics of Agitation-Induced Aggregation of Hen Lysozyme under Heat and Acidic Conditions from Various Spectroscopic Methods. PLoS ONE.

[9] Torsteinsdóttir I., Hâkansson L., Hällgren R., Gudbjörnsson B., Arvidson N.G., Venge P. (1999). Serum lysozyme: A potential marker of monocyte/macrophage activity in rheumatoid arthritis. Rheumatology.

[10] Serra C., Vizoso F., Alonso L., Rodríguez J.C., González L.O., Fernández M. (2002). Expression and prognostic significance of lysozyme in male breast cancer. Breast Cancer Res..

[11] Moghadam T., Ranjbar B. (2015). Heat induced aggregation of gold nanorods for rapid visual detection of lysozyme. Talanta.

[12] Sandin L., Nath S., Armstrong A., Janefjord C., McCann H., Halliday G.M. (2015). The role of lysozyme in Alzheimer’s disease. Alzheimers Dement..

[13] Pleyer C., Flesche J., Saeed F. (2015). Lysozyme amyloidosis—A case report and review of the literature. Clin. Nephrol. Case Stud..

[14] Pepys M.B., Hawkins P.N., Booth D.R., Vigushin D.M., Tennent G.A., Soutar A.K., Totty N., Nguyen O., Blake C.C.F., Terry C.J. (1993). Human lysozyme gene mutations cause hereditary systemic amyloidosis. Nature.

[15] Dumoulin M., Last A.M., Desmyter A., Decanniere K., Canet D., Larsson G. (2003). A camelid antibody fragment inhibits the formation of amyloid fibrils by human lysozyme. Nature.

[16] Cheng A.K., Ge B., Yu H.Z. (2007). Aptamer-Based Biosensors for Label-Free Voltammetric Detection of Lysozyme. Anal. Chem..

[17] Schneider N., Becker C.-M., Pischetsrieder M. (2010). Analysis of lysozyme in cheese by immunocapture mass spectrometry. J. Chromatogr. B.

[18] Chen H., Kong J., Yuan D., Fu G. (2014). Synthesis of surface molecularly imprinted nanoparticles for recognition of lysozyme using a metal coordination monomer. Biosens. Bioelectron..

[19] Matsunaga T., Hishiya T., Takeuchi T. (2007). Surface plasmon resonance sensor for lysozyme based on molecularly imprinted thin films. Anal. Chim. Acta..

[20] Bai Y., Zhao R., Feng F., He X. (2017). Determination of Lysozyme by Thiol-Terminated Aptamer Based Surface Plasmon Resonance. Anal. Lett..

[21] Pereira-Barros M.A., Daeid N.N., Adegoke O. (2021). Rapid and selective aptamer-based fluorescence detection of salivary lysozyme using plasmonic metal-enhanced fluorescence of ZnSSe alloyed quantum dots-gold nanoparticle nanohybrid. J. Photochem. Photobiol. A.

[22] Xie Y., An Y., Shi P., Ye N. (2017). Determination of Lysozyme by Graphene Oxide–Polyethylene Glycol-Based Fluorescence Resonance Energy Transfer. Anal. Lett..

[23] Lu L., Wang W., Wang M., Kang T.-S., Lu J.-J., Chen X.-P., Han Q.-B., Leung C.-H. (2016). A luminescent G-quadruplex-selective iridium(III) complex. J. Mater. Chem. B.

[24] Ghosh S., Khan N.I., Tsavalas J.G., Song E. (2018). Selective Detection of Lysozyme Biomarker Utilizing Large Area Chemical Vapor Deposition-Grown Graphene-Based Field-Effect Transistor. Front. Bioeng. Biotechnol..

[25] Fu X., Fu X., Wang Q., Sheng L., Huang X., Ma M., Cai Z. (2017). Fluorescence switch biosensor based on quantum dots and gold nanoparticles for discriminative detection of lysozyme. Int. J. Biol. Macromol..

[26] Wang Z., Meng Z., Xue M., Zhang H., She K.J., Kanga L. (2020). Detection of lysozyme in body fluid based on two-dimensional colloidal crystal sensor. Microchem. J..

[27] Banchelli M., Tiribilli B., Pini R., Dei L., Matteini P., Caminati G. (2016). Controlled graphene oxide assembly on silver nanocubes monolayers for SERS detection: Dependence on nanocubes packing procedure. Beilstein J. Nanotechnol..

[28] Banchelli M., Tiribilli B., de Angelis M., Pini R., Caminati G., Matteini P. (2016). Controlled Veiling of Silver Nanocubes with Graphene Oxide for Improved Surface-Enhanced Raman Scattering Detection. ACS Appl. Mater. Interfaces.

[29] Fan C., Chen H.-Y. (2014). Functional nanoprobes for ultrasensitive detection of biomolecules, Surface Enhanced Raman Spectroscopy detection. Chem. Soc. Rev..

[30] Liu S., Hu Q., Li C., Zhang F., Gu H., Wang X., Li S., Xue L., Madl T., Zhang Y. (2021). Wide-Range, Rapid, and Specific Identification of Pathogenic Bacteria by Surface-Enhanced Raman Spectroscopy. ACS Sens..

[31] Ding S.-Y., You E.-M., Tian Z.-Q., Moskovits M. (2017). Electromagnetic theories of surface-enhanced Raman spectroscopy. Chem. Soc. Rev..

[32] Ding S.-Y., Yi J., Li J.-F., Ren B., Wu D.-Y., Panneerselvam R., Tian Z.-Q. (2016). Nanostructure-based plasmon enhanced Raman spectroscopy for surface analysis of materials. Nat. Rev. Mater..

[33] Polavarapu L., Perez-Juste J., Xu Q.-H., Liz-Marzan L.M.J. (2014). Optical sensing of biological, chemical and ionic species through aggregation of plasmonic nanoparticles. Mater. Chem. C.

[34] Khlebtsov B.N., Khanadeev V.A., Panfilova E.V., Bratashov D.N., Khlebtsov N.G. (2015). Gold Nanoisland Films as reproducible SERS Substrates for Highly Sensitive Detection of Fungicides. ACS Appl. Mater. Interfaces.

[35] Li M., Cushing K., Zhou G., Wu N. (2020). Molecular hot spots in surface-enhanced Raman scattering. Nanoscale.

[36] Anker J.N., Hall W.P., Lyandres O., Shah N.C., Zhao J., Van Duyne R.P. (2008). Biosensing with plasmonic nanosensors. Nat. Mater..

[37] Babich E., Scherbak S., Asonkeng F., Maurer T., Lipovskii A. (2019). Hot spot statistics and SERS performance of self-assembled silver nanoisland films. Opt. Mater. Express.

[38] Jenkins J.A., Zhou Y., Thota S., Tian X., Zhao X., Zou S., Zhao J. (2014). Blue-Shifted Narrow Localized Surface Plasmon Resonance from Dipole Coupling in Gold Nanoparticle Random Arrays. J. Phys. Chem. C.

[39] Wen Z.Q. (2007). Raman Spectroscopy of Protein Pharmaceuticals. J. Pharm. Sci..

[40] Das G., Mecarini F., Gentile F., De Angelis F., Kumar M., Candeloro P., Liberale C., Cuda G., Di Fabrizio E. (2009). SERS on Au nanograin-aggregate structures. Biosens. Bioelectron..

[41] Wang P., Liang O., Zhang W., Schroeder T., Xie Y.H. (2013). Ultra-sensitive graphene-plasmonic hybrid platform for label-free detection. Adv. Mat..

[42] He P., Zhang Y., Liu L., Qiao W., Zhang S. (2013). Ultrasensitive SERS Detection of Lysozyme by a Target-Triggering Multiple Cycle Amplification Strategy Based on a Gold Substrate. Chem. Eur. J..

[43] Lua H., Jin M., Mac X., Yana Z., Liua Z., Wang X., Akinoglu E.M., van den Berga A., Zhou G., Shui L. (2020). Ag Nano-Assemblies on Si Surface via CTAB-Assisted Galvanic Reaction for Sensitive and Reliable Surface-Enhanced Raman Scattering Detection. Sens. Actuators B Chem..

[44] Muzi L., Seifert C., Soltani R., Ménard-Moyon C., Dumortier H., Bianco A. (2021). Targeting B Lymphocytes Using Protein-Functionalized Graphene Oxide. Adv. NanoBiomed. Res..

[45] Shan W., Pan Y., Fang H., Guo M., Nie Z., Huang Y., Yao S. (2014). An aptamer-based quartz crystal microbalance biosensor, for sensitive and selective detection of leukemia cells using silver-enhanced gold nanoparticle label. Talanta.

[46] Siekkinen A.R., McLellan J.M., Chen J., Xia Y. (2006). Rapid synthesis of small silver nanocubes by mediating polyol reduction with a trace amount of sodium sulfide or sodium hydrosulfide. Chem. Phys. Lett..

[47] Sherry L.J., Chang S., Schatz G.C., Van Duyne R.P., Wiley B.J., Xia Y. (2005). Localized Surface Plasmon Resonance Spectroscopy of Single Silver Nanocubes. Nano Lett..

[48] Gill S.C., von Hippel P.H. (1989). Calculation of protein extinction coefficient from amino acid sequence data. Anal. Biochem..

[49] Al Kayal T., Russo E., Pieri L., Caminati G., Berti D., Bucciantini M., Stefani M., Baglioni P. (2012). Interactions of lysozyme with phospholipid vesicles: Effects of vesicle biophysical features on protein misfolding and aggregation. Soft Matter.

[50] Groenning M. (2010). Binding mode of Thioflavin T and other molecular probes in the context of amyloid fibrils—Current status. J. Chem. Biol..

[51] Höök F., Rodahl M., Brzezinski P., Kasemo B. (1998). Energy Dissipation Kinetics for Protein and Antibody-Antigen Adsorption under Shear Oscillation on a Quartz Crystal Microbalance. Langmuir.

[52] Voinova M.V., Rodahl M., Jonson M., Kasemo B. (1999). Viscoelastic Acoustic Response of Layered Polymer Films at Fluid-Solid Interfaces: Continuum Mechanics Approach. Phys. Scr..

[53] Gambinossi F., Banchelli M., Durand A., Berti D., Brown T., Caminati G., Baglioni P. (2010). Modulation of Density and Orientation of Amphiphilic DNA Anchored to Phospholipid Membranes. I. Supported Lipid Bilayers. J. Phys. Chem. B.

[54] Panfilova E.V., Khlebtsov B.N., Burov A.M., Khlebtsov N.G. (2012). Study of Polyol Synthesis Reaction Parameters Controlling High Yield of Silver Nanocubes. Colloid J..

[55] Mittal R., Glenn R., Saytashev I., Lozovoy V.V., Dantus M. (2015). Femtosecond Nanoplasmonic Dephasing of Individual Silver Nanoparticles and Small Clusters. J. Phys. Chem. Lett..

[56] Bhattacharya R., Basu J.K.J. (2013). Microscopic dynamics of nanoparticle monolayers at air-water interface. Coll. Interf. Sci..

[57] Roberts G. (1990). Langmuir-Blodgett Films.

[58] Bottomley A., Prezgot D., Staff A., Ianoul A. (2012). Fine tuning of plasmonic properties of monolayers of weakly interacting silver nanocubes on thin silicon films. Nanoscale.

[59] Gao Y., Zhang R., Cheng J.-C., Liaw J.-W., Ma C. (2013). Optical properties of plasmonic dimer, trimer, tetramer and pentamer assemblies of gold nanoboxes. J. Quant. Spectrosc. Radiat. Transf..

[60] Toma M., Toma K., Michioka K., Ikezoe Y., Obara D., Okamoto K., Tamada K. (2011). Collective plasmon modes excited on a silver nanoparticle 2D crystalline sheet. Phys. Chem. Chem. Phys..

[61] Aboutalebi S.H., Gudarzi M.M., Zheng Q.B., Kim J.-K. (2011). Spontaneous formation of liquid crystals in Ultralarge Graphene oxide dispersions. Adv. Funct. Mater..

[62] Dan B., Behabtu N., Martinez A., Evans J.S., Kosynkin D.V., Tour J.M. (2011). Liquid crystals of aqueous, giant graphene oxide flakes. Soft Matter.

[63] McCoy T.M., de Campo L., Sokolova A.V., Grillo I., Izgorodina E.I., Tabor R.F. (2018). Bulk properties of aqueous graphene oxide and reduced graphene oxide with surfactants and polymers: Adsorption and stability. Phys. Chem. Chem. Phys..

[64] Zhu Y. (2010). Graphene and Graphene Oxide: Synthesis, Properties, and Applications. Adv. Mater..

[65] Chandrasekaran N., Dimartino S., Fee C.J. (2013). Study of the adsorption of proteins on stainless steel surfaces using QCM-D. Chem. Eng. Res. Des..

[66] Wang X., Herting G., Odnevall Wallinder I., Blomberg E. (2014). Adsorption of Lysozyme on Silver and Its Influence on Silver Release. Langmuir.

[67] Chandra G., Ghosh K.S., Dasgupta S., Roy A. (2010). Evidence of conformational changes in adsorbed lysozyme molecule on silver colloids. Int. J. Biol. Macromol..

[68] Xu K.R., Ouberai M.M., Welland M.E. (2013). A comprehensive study of lysozyme adsorption using dual polarization interferometry and quartz crystal microbalance with dissipation. Biomaterials.

[69] Blake C.C.F., Koenig D.F., Mair G.A., North A.C.T., Phillips D.C., Sarma V.R. (1965). Structure of hen egg-white lysozyme—A 3-dimensional Fourier synthesis at 2 angstrom resolution. Nature.

[70] Kubiak-Ossowska K., Mulheran P.A. (2010). Mechanism of hen egg white lysozyme adsorption on a charged solid surface. Langmuir.

[71] Bingen P., Wang G., Steinmetz N.F., Rodahl M., Richter R.P. (2008). Solvation effects in the quartz crystal microbalance with dissipation monitoring response to biomolecular adsorption. A phenomenological approach. Anal. Chem..

[72] Reymond-Laruinaz S., Saviot L., Potin V., del Carmen M., de Lucas M. (2016). Protein–nanoparticle interaction in bioconjugated silver nanoparticles: A transmission electron microscopy and surface enhanced Raman spectroscopy study. Appl. Surf. Sci..

[73] Haynes C.A., Norde W. (1994). Globular proteins at solid/liquid interfaces. Colloids Surf. B.

[74] Henzler K., Haupt B., Lauterbach K., Wittemann A., Borisov O., Ballauff M. (2010). Adsorption of β-Lactoglobulin on Spherical Polyelectrolyte Brushes: Direct Proof of Counterion Release by Isothermal Titration Calorimetry. J. Am. Chem. Soc..

[75] Vasilescu A., Gáspár S., Gheorghiu M., David S., Dinca V., Peteu S., Wang Q., Li M., Boukherroub R., Szunerits S. (2017). Surface Plasmon Resonance based sensing of lysozyme in serum on Micrococcus lysodeikticus modified graphene oxide surfaces. Biosens. Bioelectron..

[76] Baweja L., Balamurugan K., Subramanian V., Dhawan A. (2015). Effect of graphene oxide on the conformational transitions of amyloid beta peptide: A molecular dynamics simulation study. J. Mol. Graph. Model..

[77] Malik S.A., Mohanta Z., Srivastava C., Atreya H.S. (2020). Modulation of protein–graphene oxide interactions with varying degrees of oxidation. Nanoscale Adv..

[78] Şengül Ü. (2016). Comparing determination methods of detection and quantification limits for aflatoxin analysis in hazelnut. J. Food Drug Anal..

[79] Ye S., Xiao J., Guo Y., Zhang S. (2013). Aptamer-Based SERS Assay of ATP and Lysozyme by Using Primer Self-Generation. Chem. Eur. J..

[80] Zhang L., Lian W., Li P., Ma H., Han X., Zhao B., Chen Z. (2020). Crocein Orange G mediated detection and modulation of amyloid fibrillation revealed by surface-enhanced Raman spectroscopy. Biosens. Bioelectron..

[81] Krimm S., Bandekar J. (1986). Vibrational Spectroscopy and Conformation of Peptides, Polypeptides, and Proteins. Adv. Protein Chem..

[82] Kuhar N., Sil S., Umapathy S. (2021). Potential of Raman spectroscopic techniques to study proteins. Spectrochim. Acta A Mol. Biomol. Spectrosc..

[83] Mahmoud M.A., Tabor C.E., El-Sayed M.A. (2009). Surface-Enhanced Raman Scattering Enhancement by Aggregated Silver Nanocube Monolayers Assembled by the Langmuir−Blodgett Technique at Different Surface Pressures. J. Phys. Chem. C.

[84] Dong A.C., Huang P., Caughey W.S. (1990). Protein Secondary Structures in Water from Second-Derivative Amide I Infrared Spectra. Biochemistry.

[85] Lippert J.L., Lindsay R.M., Schultz R. (1980). Laser-Raman investigation of lysozyme-phospholipid interactions. Biochim. Biophys. Acta.

[86] Brandt E.-S., Cotton T.M., Rossiter B.W., Baetzold R.C. (1993). Surface-Enhanced Raman Scattering. Investigations of Surface and Interfaces.

[87] Podstawka E., Ozaki Y., Proniewicz L.M. (2004). Adsorption of S-S Containing Proteins on a Colloidal Silver Surface Studied by Surface-Enhanced Raman Spectroscopy. Appl. Spectrosc..

[88] Agarwal N.R., Tommasini M., Ciusani E., Lucotti A., Trusso S., Ossi P.M. (2018). Protein-Metal Interactions Probed by SERS: Lysozyme on Nanostructured Gold Surface. Plasmonics.

[89] Hu J., Sheng R.S., Xu Z.S. (1995). Surface enhanced Raman spectroscopy of lysozyme. Spectrochim. Acta.

[90] Mdluli P.S., Sosibo N.M., Revaprasadu N., Karamanis P., Leszczynski J. (2009). Surface enhanced Raman spectroscopy (SERS) and density functional theory (DFT) study for understanding the regioselective adsorption of pyrrolidinone on the surface of silver and gold colloids. J. Mol. Struct..

[91] Kurouski D., Van Duyne R.P., Lednev I.K. (2015). Exploring the structure and formation mechanism of amyloid fibrils by Raman spectroscopy: A review. Crit. Rev. Anal..

[92] Huang H.H., Ni X.P., Loy G.L., Chew C.H., Tan K.L., Loh F.C., Deng J.F., Xu G.Q. (1996). Photochemical Formation of Silver Nanoparticles in Poly(*N*-vinylpyrrolidone). Langmuir.

[93] Shang Z., Li L., Zhang D., Wang C.-E., Tang Z., Zou M., Gong H., Yu Z., Jin S., Liang P. (2021). Competitive adsorption of residual polyvinylpyrrolidone and detection molecular on flower liked silver nanoparticles. Spectrochim Acta A Mol. Biomol. Spectrosc..

[94] Dhafer C.E., Mezni A., Smiri L.S. (2017). Surface-enhanced Raman scattering study of Ag-PVP interactions in the biocompatible Ag@PVP nanoparticles. J. Tun. Chem. Soc..

[95] Morales-Narváez E., Arben M. (2018). Graphene Oxide as an Optical Biosensing Platform: A Progress Report. Adv. Mater..

[96] Moretti M., Allione M., Marini M., Torre B., Giugni A., Limongi T., Das G., Di Fabrizio E. (2017). Raman study of lysozyme amyloid fibrils suspended on super-hydrophobic surfaces by shear flow. Microelectron. Eng..

[97] Chiti F., Dobson C.M. (2009). Amyloid formation by globular proteins under native conditions. Nat. Chem. Biol..

[98] Chen F., Cai C., Chen X., Chen C. (2016). “Click on the bidirectional switch”: The aptasensor for simultaneous detection of lysozyme and ATP with high sensitivity and high selectivity. Sci. Rep..

[99] Arabzadeh A., Salimi A. (2015). Novel voltammetric and impedimetric sensor for femtomolar determination of lysozyme based on metal–chelate affinity immobilized onto gold nanoparticles. Biosens. Bioelectron..

[100] Sener G., Ozgur E., Yılmaz E., Uzuna L., Sayc R., Denizli A. (2010). Quartz crystal microbalance based nanosensor for lysozyme detection with lysozyme imprinted nanoparticles. Biosens. Bioelectron..

[101] Subramanian P., Lesniewski A., Kaminska I., Vlandas A., Vasilescu A., Niedziolka-Jonsson J., Pichonatc E., Happy H., Boukherrouba R., Szunerits S. (2013). Lysozyme detection on aptamer functionalized graphene-coated SPR interfaces. Biosens. Bioelectron..

[102] Vidal M.-L., Gautron J., Nys Y. (2005). Development of an ELISA for Quantifying Lysozyme in Hen Egg White. J. Agric. Food Chem..

[103] Mihai I., Vezeanua A., Polonschii C., Albub C., Radub G.-L., Vasilescu A. (2015). Label-free detection of lysozyme in wines using an aptamer based biosensor and SPR detection. Sens. Actuators B.

[104] Fan C., Chen Z.-Q., Li C., Wang Y.-L., Yu Q., Zhu M.-Q. (2021). Hydrophilic AIE-Active Tetraarylethenes for Fluorescence Sensing and Super-Resolution Imaging of Amyloid Fibrils from Hen Egg White Lysozyme. ACS Appl. Mater. Interfaces.

[105] Vasilescu A., Gaspar S., Mihai I., Tacheb A., Litescub S.C. (2013). Development of a label-free aptasensor for monitoring the self-association of lysozyme. Analyst.

[106] Sener G., Uzun L., Say R., Denizli A. (2011). Use of molecular imprinted nanoparticles as biorecognition element on surface plasmon resonance sensor. Sens. Actuators B.

[107] Rodríguez M.C., Rivas G.A. (2009). Label-free electrochemical aptasensor for the detection of lysozyme. Talanta.

[108] Kocherbitov V., Latynis J., Misiūnas A., Barauskas J., Niaura G. (2013). Hydration of Lysozyme Studied by Raman Spectroscopy. J. Phys. Chem. B.

