# Supplementary material for: Graphene Oxide/Silver Nanoparticles Platforms for the Detection and Discrimination of Native and Fibrillar Lysozyme: A Combined QCM and SERS Approach
Source: Nanomaterials (Basel). 2022 Feb 10;12(4):600. doi: 10.3390/nano12040600 (PMC8878839; doi:10.3390/nano12040600)
Supplement: Supplementary file 1 [file nanomaterials-12-00600-s001.zip › nanomaterials-1559881-supplementary.pdf]

Supplementary Materials for

Graphene Oxide/Silver Nanoparticles Platforms for the Detection and Discrimination of Native and Fibrillar Lysozyme: A Combined QCM and SERS Approach

Vania Tramonti, Cristiana Lofrumento, Maria Raffaella Martina, Giacomo Lucchesi and Gabriella Caminati *

Department of Chemistry and CSGI, University of Florence, Via della Lastruccia 3-13,
50019 Sesto Fiorentino, Italy; vania.tramonti@gmail.com (V.T.); cristiana.lofrumento@unifi.it (C.L.); martinamariella@gmail.com (M.R.M.); giacomo.lucchesi@unifi.it (G.L.)
* Correspondence: gabriella.caminati@unifi.it

Preparation of Fibrillar HWL

Previous studies have shown that lysozyme can easily form amyloid fibrils under different conditions, such as high temperatures and appropriate pH conditions [6–8,97]. We used these established protocols and followed the formation of HWL fibrils monitoring the increase of ThT fluorescence emission at 450 nm and 480 upon excitation at 330 nm and 450 nm, respectively.

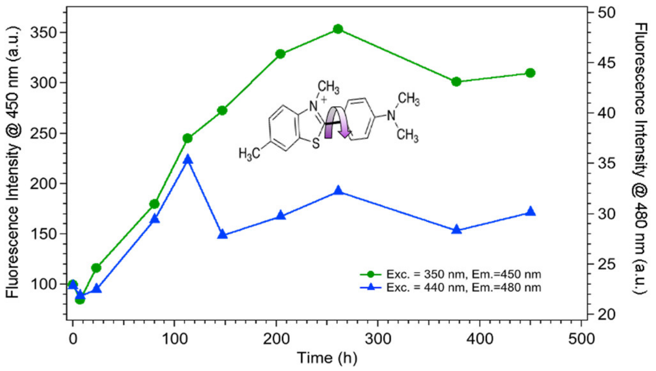

Figure S1. THT fluorescence emission at 480 nm (blue line) and 450 nm (green line) as a function of time of incubation.

The presence of amyloid fibrils at the end of the aggregation process was confirmed by Circular Dichroism spectroscopy. Figure S2 shows the spectra obtained for lysozyme initial solution and lysozyme after 450 h incubation in acidic solution at 60 °C.

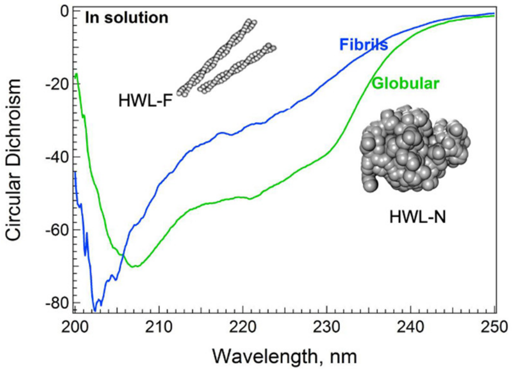

Figure S2. Circular Dichroism spectra for lysozyme solution along the aggregation process, samples taken at t = 0 and t = 450 h.

TEM image of AgNC/NS

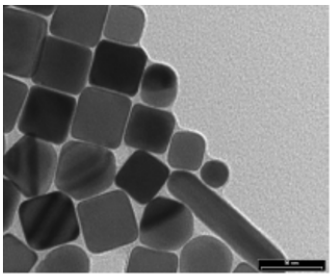

Figure S3. TEM image of the AgNC/NS nanoparticles. Scale bar = 50 nm

Absorption Spectra of GO

UV-Vis spectra of GO-AgNC/NS prepared on quartz slides showed a strong absorption peak at 227.5 nm in the ultraviolet-visible spectrum attributed to the π-π transition of the aromatic C–C bonds of graphene oxide in agreement with literature reports [5]

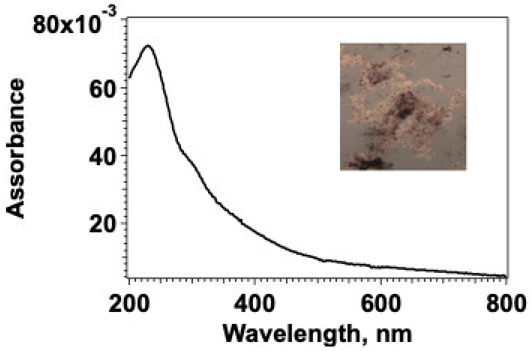

Figure S4. Absorption spectrum of GO-AgNC/NS metasurface prepared on quartz slides. Inset: optical microscope images the GO-AgNC/NS metasurface.

Kinetics of Adsorption of HWL-N and HWL-F on AgNC/NS and GO_AgNC/NS

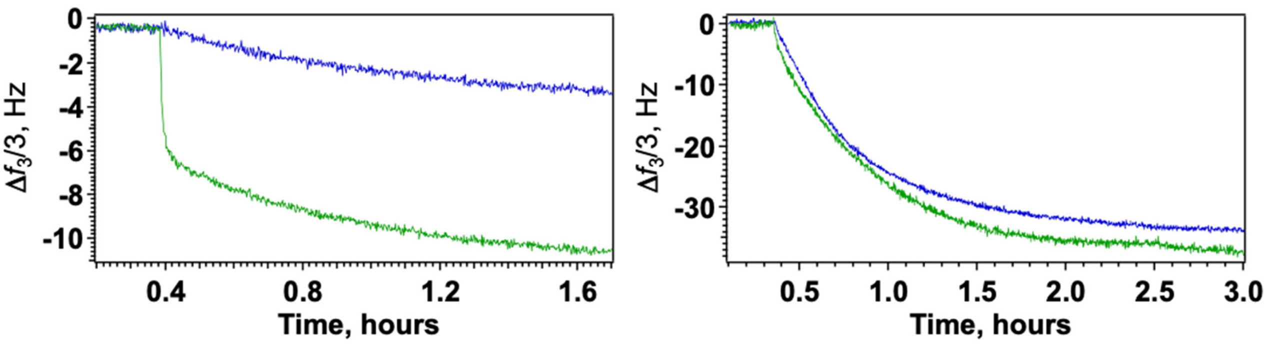

(a) (b)

Figure S5. (a) Kinetics of adsorption of HWL-N (blue) and HWL-F (green) on bare AgNC/NS arrays. (b) Kinetics of adsorption of HWL-N (blue) and HWL-F (green) on GO-AgNC/NS arrays. Concentration of lysozyme = 1 × 10^{-6} M in all cases.

Table S1. Fitting parameters of the kinetic curves of adsorption of HWL-N and HWL-F on different nanosensor platforms

	**HWL-N**		**HWL-F**	
	**t_1, h (A_1)**	**t_2, h (A_2)**	**t_1, h (A_1)**	**t_2, h (A_2)**
**Nanoplatform**				
**AgNC**	0.7823 ± 0.0366	-	0.0134 ± 0.0006 (7.051 ± 0.198)	0.6176 ± 0.0273 (4.892 ± 0.062)
**GO/AgNC**	0.5014 ± 0.0033	-	0.54441 ± 0.00365	-

Cartoon of HWL-N

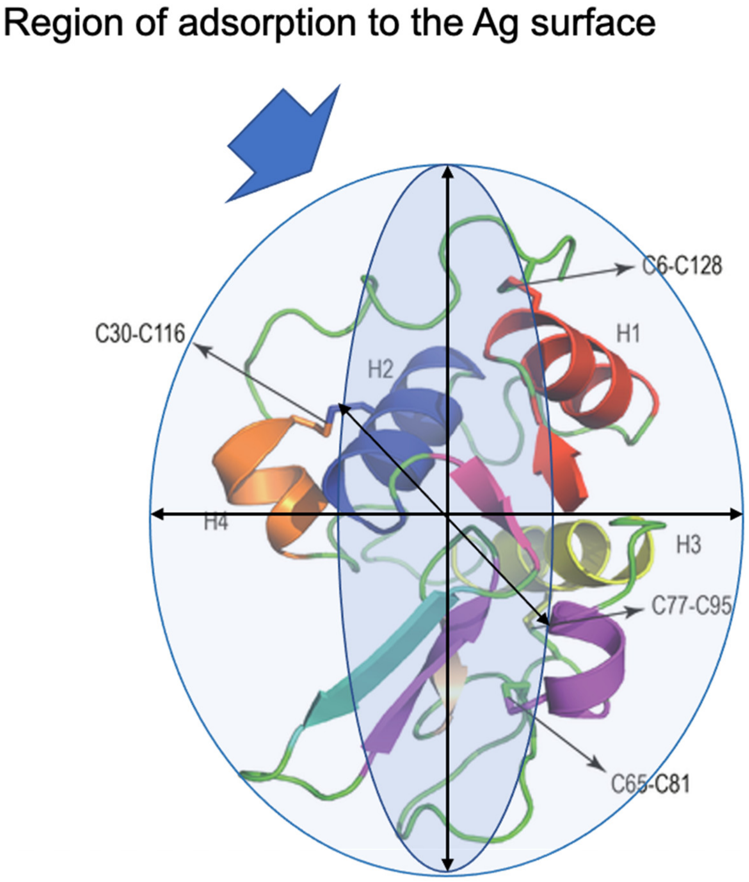

Figure S6. Native lysozyme conformation

Confocal Laser Scanning Microscopy of Aged Samples of HWL-N Adsorbed onto AgNC/NS Arrays.

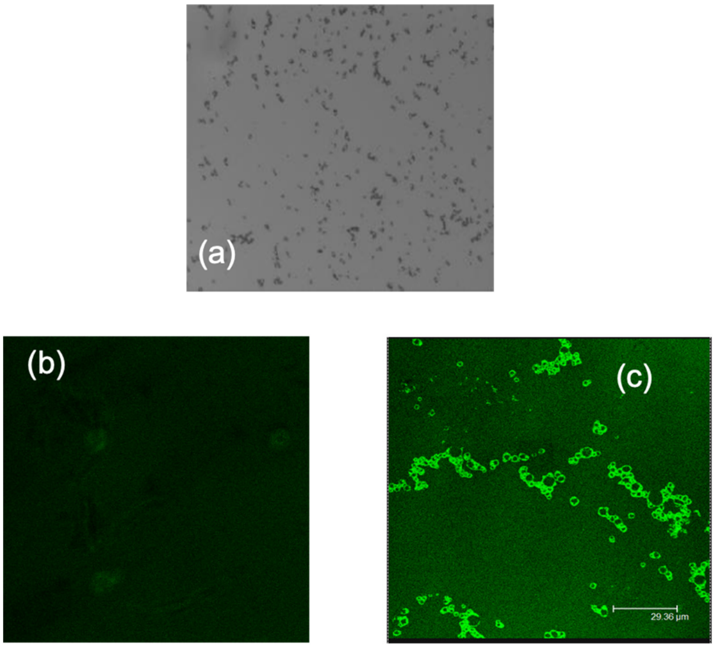

Figure S7. Phase contrast (a) and Confocal Laser Scanning Microscopy images of HWL-N adsorbed on AgNC/NS at t = 0 (b) and t = 48 h (c). ThT was added prior to CLSM observation.

When the native lysozyme, HWL-N, sample is left aging on the naked AgNC/NS nanostructured platforms a progressive transition to aggregated amyloid forms of protofibrils is clearly observed as reported by the appearance of the ThT fluorescent fingerprint of beta-amyloid conformation. Aggregation occurs at the edges of the AgNC/NS clusters likely triggered by localized adsorption as shown in Figure S7 (c).

Calibration Curve for Native and Fibrillar HWL on GO-AgNC/NS

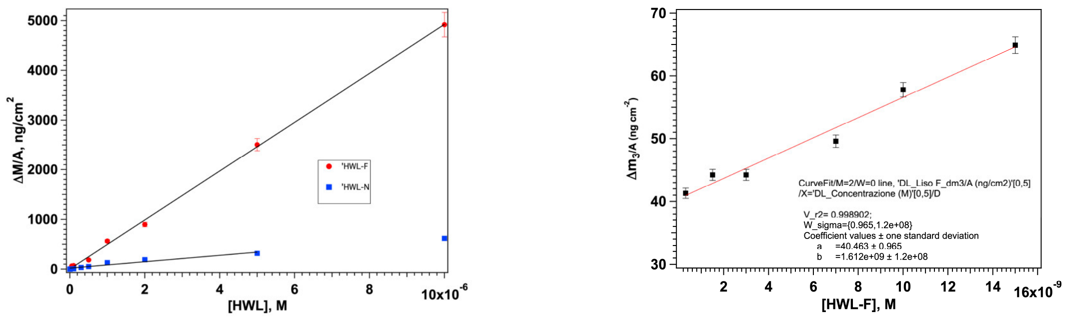

(a) **(b)**

Figure S8. (a) Adsorbed surface mass density as a function of bulk concentration for spiked samples of HWL-N (blue squares) and HWL-F (red dots) on GO-AgNC/NC sensors. Solid lines are the linear regression fit. (b) Calibration curve obtained for HWL-F for monolayers of fibrils (concentration regime 0–1.5 × 10^{-8} M).

Table S2. Linear regression fit parameters and LOD

	Concentration range, M	R^2	LOD, nM
HWL-N	0–1 × 10^{-6}	0.999287	14.8
HWL-F	0–1 × 10^{-5}	0.999109	154.8
	0–1 × 10^{-8}	0.989207	1.9

Survey of Recent Literature on Lysozyme Detection

Table S3. Summary of relevant studies on native lysozyme detection.

METHOD	PLATFORM	LOD	Linearity range	References
Voltammetric Detection	Aptamer-Based	35 nM<br>0.5 µg/mL		[16]
Immunocapture mass spectrometry		5 mg/kg		[17]
Surface plasmon resonance sensor	Molecularly imprinted thin films	-		[19]
Surface Plasmon Resonance	Thiol-Terminated-Aptamer-based biosensor	35 nM<br>0.5 µg/mL	1–100 µM	[20]
Fluorescence	Quantum dots/Aptamer-based	1.6 µM<br>22.9 µg/mL		[21]
FRET	Graphene Oxide–Polyethylene Glycol	11 nM<br>0.16 µg/mL	50–300 nM	[22]
Fluorescence	Quantum dots and gold nanoparticles	2.3 nM<br>33.43 ng/mL	50–1000 ng/mL	[25]
Debye diffraction ring diameter.	Colloidal crystal arrays	96 nM<br>1.38 µg/mL		[26]
Fluorescence	Aptamer based	1 pM<br>14 pg/mL		[98]
Electrochemical Impedance Spectroscopy (EIS)/ Voltammetry	Metal-chelate Gold nanoparticles	60 fM<br>0.86 pg/mL<br>80 fM<br>1.2 pg/mL	0.1 mM<br><br>(0.1 pM to 0.10 mM	[99]
Quartz Crystal Microbalance	Molecular imprinted nanoparticles	83 pM<br>1.2 ng/mL;	0.2–100 µg/L	[100]
Surface Plasmon Resonance	Anti-lysozime DNA aptamer	0.5 nM<br>7.2 ng/mL	0.5–200 nM	[101]
Fluorescence	Indirect-ELISA	18 nM<br>0.264 µg/mL	0.38–4.8 µg/mL	[102]
Size exclusion chromatograpy with fluorimetric detection	Aptamer -based	35 nM<br>0.5 µg/mL		[103]
Surface Plasmon Resonance	Aptamer -based	2.44 nM<br>35 ng/mL	0.05–1 µg/mL	[103]

Table S4. Summary of relevant studies on fibrillar lysozyme detection.

METHOD	PLATFORM	LOD	Linearity range	References
Fluorescence	Solution	0.59 nM<br>8.5 ng/mL	0–10 μg/mL	[104]
SPR<br>Size-exclusion chromatography (SEC) and Matrix Assisted Laser Desorption Ionization Time of Flight Mass Spectrometry (MALDI-TOF-MS).	Aptamer-based	70 nM<br>1 μg/mL	5–50 μg/mL	[105]
SPR	Molecular Imprinting	32.2 nM<br>0.46 μg/mL	-	[106]
Electrochemical	Aptamer-based	36 nM<br>0.52 μg/mL	-	[107]

SERS Samples on quartz slides and gold coated QCM sensors

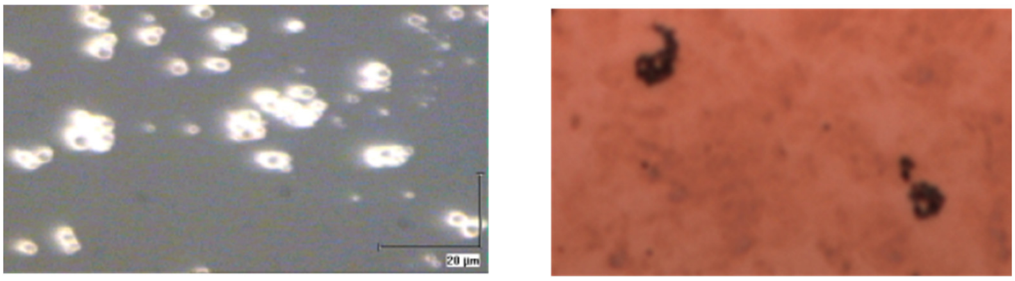

(a) **(b)**

Figure S9. Optical microscopy in reflection mode of SERS samples prepared on quartz substrates (a) and QCM gold-coated sensors (b).

Summary of the principal Raman bands for HWL-N and HWL-F

Table S5. Assignment of the main peaks observed in SERS spectra

	Peak frequency (cm^{-1})		Assignment	References
**Molecular group**	**HWL-F**	**HWL-N**		
**Tyr**	373			[89]
**Cys**	504	510	ν(S – S)	[87,108]
**Phe/Cys**		623	in-plane ring deformation, ν(C – S) of Cysteine	[83,108]
**Tyr/Cys**		644	ν(C – S) of Cysteine	[40,83]
**Trp**	758		indole ring breathing	[40,87,108]
**His**	772			[40,108]
**Cys/ OH group of Tyr**		845		[89]
**Trp**	875		Benzene ring breathing + δ(N – H), W17 mode	[108]
**N - C_α – C main chain**	924		ν(N - C_α – C)	[67,108]
**Phe**	1005	1005	Ring breathing	[108]
**Phe**	1075	1072	ν(C-N)	[40,108]
**Trp**	1132	1132	Ring/ ν(C-N)	[87,89]
**Amide III**	1239	1239 (double peak)	β-sheet	[89,108]
**Amide III**	1299		α-helix	[40,67]
**Amide**	1329			[40,67]
	1396 (intense)	1396	ν(COO)	[89]
		1423		[89]
		1441		[89]
	1459 (intense)	1459	CH deformation	[108]
**Trp**	1542		Indole ring stretching/amide II	[108]
**Phe**	1577		Ring breathing	[108]
**Tyr**		1613		[83,87]
**Amide I**	1665		organized β-sheet structure	[40,83]

Dependence of SERS spectra from excitation wavelength

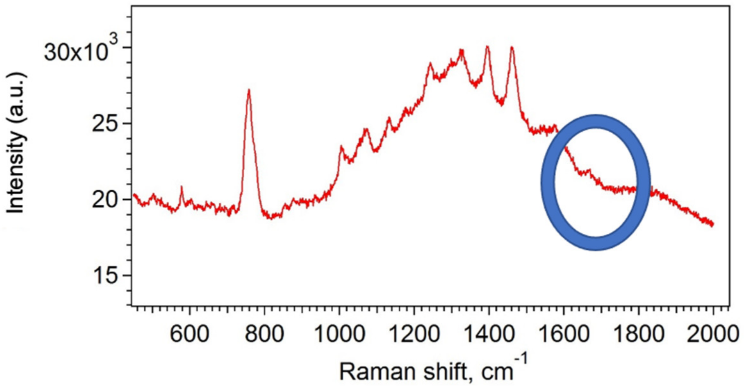

Figure S10. SERS spectrum for HWL-F adsorbed on GO-AgNC/NS, excitation at 532 nm.

SERS spectra in the 180–400 cm^{-1} spectral range

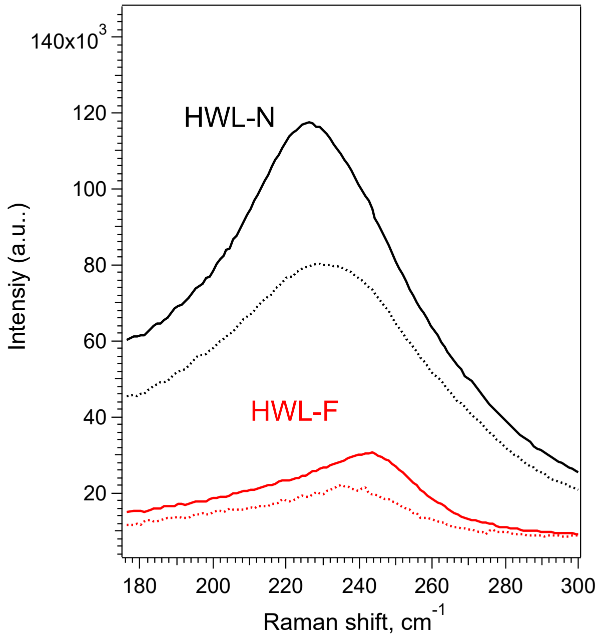

Figure S11. SERS spectra in the 180–300 cm–^{1} spectral range. Solid lines: protein adsorbed on GO-AgNC/NS, dotted line: protein adsorbed on AgNC/NS. [lysozyme]=1 × 10^{-6} M
